# An alginate-confined peroxygenase-CLEA for styrene epoxidation†

**DOI:** 10.1039/d1cc01868j

**Published:** 2021-05-07

**Authors:** Friederike E. H. Nintzel, Yinqi Wu, Matteo Planchestainer, Martin Held, Miguel Alcalde, Frank Hollmann

**Affiliations:** Department of Biotechnology, Delft University of Technology, van der Maasweg 9 2629 HZ Delft The Netherlands f.hollmann@tudelft.nl; Department of Biosystems Science and Engineering, ETH Zürich, Mattenstrasse 26 Basel 4058 Switzerland; Department of Biocatalysis, Institute of Catalysis and Petrochemistry (CSIC) Madrid Spain

## Abstract

Oxyfunctionalisation reactions in neat substrate still pose a challenge for biocatalysis. Here, we report an alginate-confined peroxygenase-CLEA to catalyse the enantioselective epoxidation of *cis*-β-methylstyrene in a solvent-free reaction system achieving turnover numbers of 96 000 for the biocatalyst and epoxide concentrations of 48 mM.

## Abstract 2

An alginate-confined peroxygenase-CLEA has been prepared for the stereoselective epoxidation of *cis*-β-methylstyrene under non-aqueous reaction conditions. Product titres of up to 48 mM and excellent enzyme turnovers of 96 000 have been achieved.

## Graphical abstract

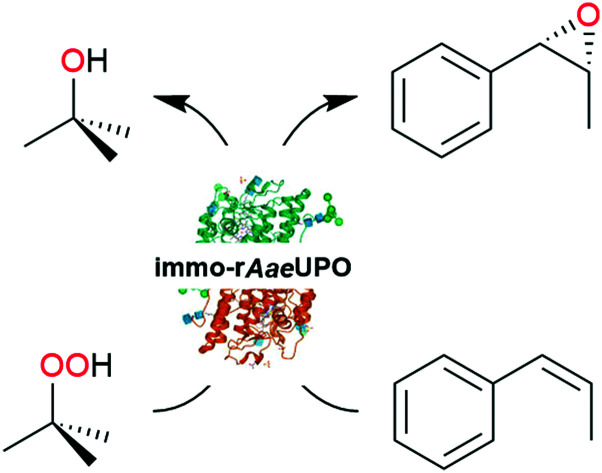

Biocatalytic oxyfunctionalisation reactions are enjoying an increasing interest in organic chemistry.^1^ Especially the often very high regio- and enantioselectivity of enzymatic oxyfunctionalisation reactions such as hydroxylations or epoxidations offers synthetic chemists straightforward access to chiral building blocks, which with traditional chemical means are difficult to prepare.

Next to the well-known P450 monooxygenases,^2^ in recent years also peroxygenases^3^ have been in the centre of attention. P450 monooxygenases reductively activate molecular oxygen to form the catalytically active oxyferryl-heme species. Peroxygenases form this species directly from partially reduced oxygen species (peroxides) and thereby circumvent the complex molecular architectures of P450 monooxygenases (Scheme 1).

**Scheme 1:**
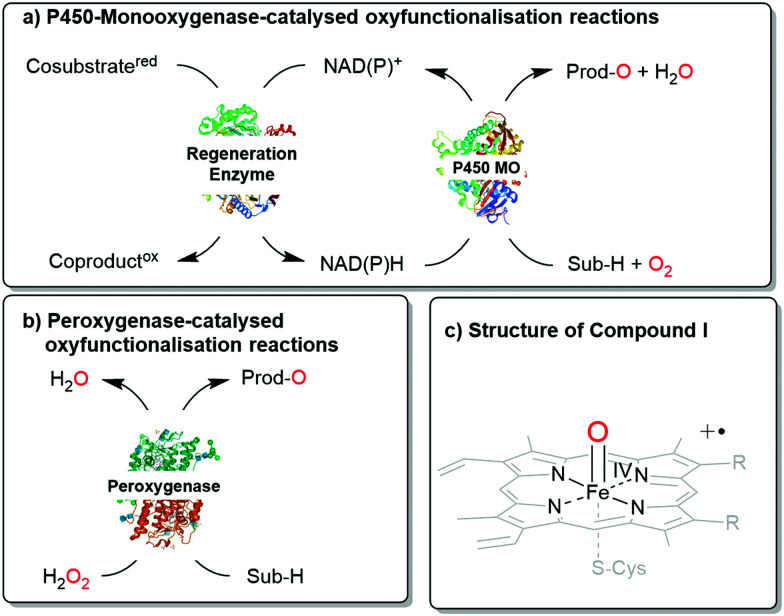
Comparison of (a) P450 monooxygenase-catalysed and (b) peroxygenase-catalysed oxyfunctionalisation reactions. (c) Both enzyme classes utilise Compound **I** as the oxygenating agent.

The synthetic application of both enzyme classes, however, still suffers from the poor water solubility of the majority of starting materials, resulting in rather dilute reaction mixtures with an enormous water footprint. Therefore, increasing the starting material (and product) concentration is of utmost importance to increase the economic viability and environmental friendliness of such biocatalytic reactions.^4^

One common approach to increase the substrate loading is to use the so-called two liquid phase approach^5^ in which an aqueous, biocatalyst-containing layer is contacted with a hydrophobic, organic layer serving as substrate reservoir and product sink. To alleviate possible phase transfer rate limitations of this system, intensive mechanical stirring is needed, which however, also may impair the stability of the biocatalyst.^6^

The latter issue can be addressed by immobilising the biocatalyst to a heterogeneous carrier material and thereby physically protecting the enzyme. While a limited number of studies report immobilisation of peroxygenases, this technique is not fully explored yet for this enzyme class.^7–12^ In previous works, we could demonstrate that immobilised peroxygenases in principle can even be applied in neat (*i.e.* almost water-free) reaction media.^9^ A drawback of this approach, however, was the very poor specific activity of the immobilised enzyme, possibly due to a combination of activity losses of the enzyme during immobilisation and further activity losses originating from dehydratation of the enzyme surface.

Encapsulating enzymes in alginate matrices may represent an elegant compromise. The so-confined enzymes are mechanically stabilised while still situated in a micro-aqueous environment.^8,11,13^

We therefore set out to immobilise a peroxygenase in an alginate matrix and evaluate its catalytic activity under non aqueous reaction conditions. As model enzyme we chose the recombinantly expressed, evolved peroxygenase from *Agrocybe aegerita* (r*Aae*UPO)^14–16^ as catalyst for the epoxidation of styrene and *cis*-β-methylstyrene (Scheme 2). The biocatalyst was obtained from the supernatant of the fermentation broth of recombinant *Pichia pastoris* and used without further purification.

**Scheme 2:**
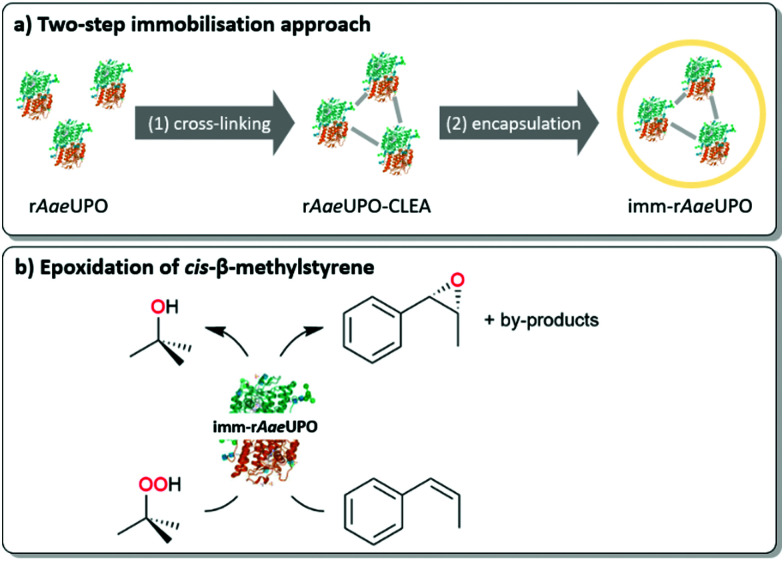
(a) Overview of the utilised immobilisation approach consisting of CLEA formation and alginate confinement. (b) Epoxidation of *cis*-β-methylstyrene by immobilised r*Aae*UPO with *tert*-butyl hydroperoxide (^*t*^BuOOH) as oxidant.

Confining r*Aae*UPO in Ca^2+^-hardened alginates proved to be feasible. To our surprise, however, the resulting immobilisate showed low, and somewhat irreproducible catalytic activity (Fig. 1a), which most likely was due to leaching of the enzyme from the beads during the immobilisation procedure and storage. To improve the retention of the biocatalyst in the alginate beads, we decided to increase its molecular mass by covalent cross-linking (CLEA formation).^17–19^

**Fig. 1:**
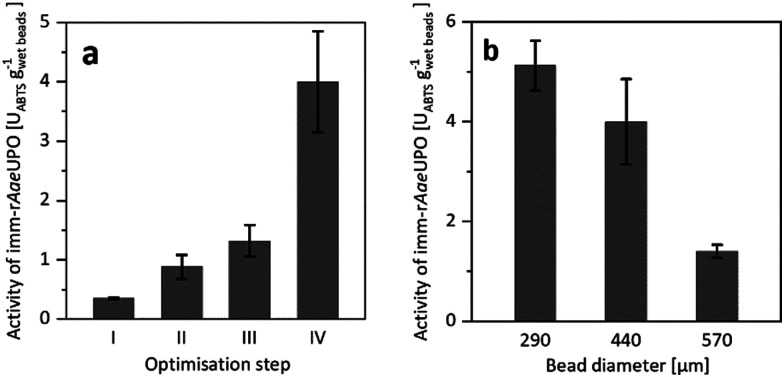
(a) Activity of imm-r*Aae*UPO during optimisation of the immobilisation procedure, determined by ABTS-activity assay in aqueous environment. Reference (I) is the encapsulation of free r*Aae*UPO. The catalytic activity of imm-r*Aae*UPO was improved by CLEA formation (II), by application of chitosan as coagulant (III), and by maximising the enzyme load (IV). (b) Activity of imm-r*Aae*UPO immobilisates with different diameters, determined by ABTS-activity assay in aqueous environment. Data represents the average of duplicates. Further information on the immobilisation optimisation can be found in the ESI.†

Indeed, CLEA formation more than doubled the catalytic activity of the immobilised peroxygenase (Fig. 1a). Further improvements were achieved by using chitosan as coagulant^20–23^ and by increasing the enzyme load (Fig. 1a). Additional optimisation steps are reported in the ESI.† It is important to note that the size of the beads had a significant influence on the activity of the immobilised peroxygenase. The larger the beads, the lower the catalytic activity under otherwise identical conditions (Fig. 1b).

Overall, approximately 19% of the enzyme was immobilised (as determined *via* quantification of the amount of active heme sites using CO-differential spectra) and 11.4% of the peroxidase activity, as judged by the ABTS oxidation activity, was found back in the immobilisates. It is worth mentioning here that r*Aae*UPO immobilisation also increased its storage stability. While the free enzyme completely lost its catalytic activity after 12 days storage at room temperature, the immobilised version exhibited at least 80% of its initial activity even after two weeks (Fig. S10, ESI†).

Having the immobilised r*Aae*UPO preparation at hand, we decided to compare its catalytic performance in the epoxidation of *cis*-β-methylstyrene with the free enzyme in a two liquid phase approach (Fig. 2) using ^*t*^BuOOH as oxidant.

**Fig. 2:**
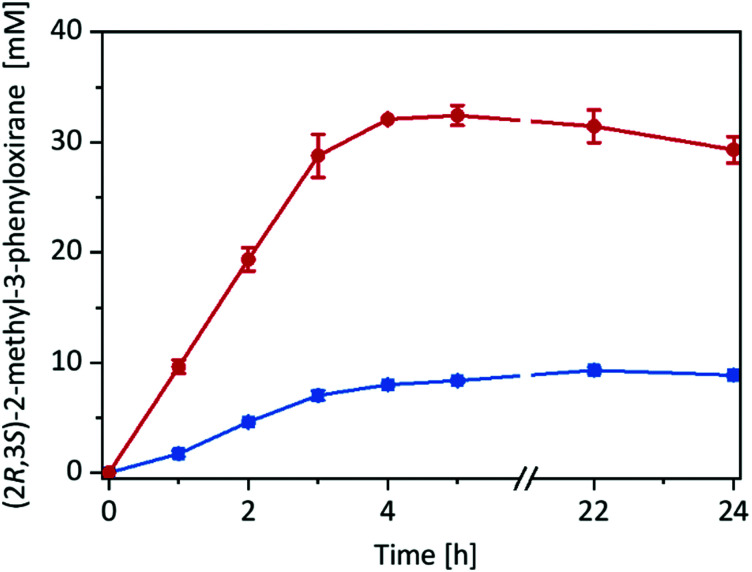
Product formation over time for the epoxidation of *cis*-β-methylstyrene in different reaction systems: imm-r*Aae*UPO in neat substrate (180 mg immobilisate in 570 μL *cis*-β-methylstyrene) (red circles), and free r*Aae*UPO in a two-liquid phase system (180 μL TRIS-HCl buffer (20 mM, pH 7): 570 μL *cis*-β-methylstyrene) (blue squares). General reaction conditions: [r*Aae*UPO] = 0.5 μM, ^*t*^BuOOH feeding rate = 10 mM h^−1^, room temperature, shaking at 99 rpm with 60° angle in an overhead rotator. Data represents the average of duplicates.

Very much to our surprise the immobilised enzyme outperformed the free enzyme under otherwise identical conditions (such as volumetric ratio of aqueous or alginate volume to the organic phase and enzyme concentrations). With the immobilised enzyme the product accumulation rate was approx. 10 mM h^−1^ (corresponding to the ^*t*^BuOOH feed rate) while with the free enzyme it was only 3 mM h^−1^. A plausible explanation for this may be the higher surface area of the reactions using alginate-immobilised r*Aae*UPO, largely eliminating the diffusion rate limitation of ^*t*^BuOOH and/or *cis*-β-methylstyrene into the aqueous reaction phase (Fig. S15, ESI†).

Overall, in this experiment, approx. 30 mM of enantiomerically pure (2*R*,3*S*)-2-methyl-3-phenyloxirane has been synthesised within 4 h corresponding to a turnover number (TN = mol_Product_ × mol_r*Aae*UPO_^−1^) for the enzyme of 60 000 and an average (over 4 h) turnover frequency of 4.1 s^−1^. It should be mentioned here, that under these reaction conditions several side products such as benzaldehyde and phenylacetone were observed (*vide infra*).

Despite the promising results, the reactions stopped after approximately 5 h. We suspected the irreversible, oxidative inactivation of the heme-containing biocatalyst by the hydroperoxide to account for the low robustness of the reaction.

Therefore, we performed a series of experiments varying the ^*t*^BuOOH addition rate (Fig. 3). While the initial product formation rate decreased with decreasing ^*t*^BuOOH feeding rates, the long-term robustness of the reaction increased: at a ^*t*^BuOOH feeding rate of 20 mM h^−1^, product formation rates of 9 mM h^−1^ were observed but the product accumulation ceased after 4 h. Applying a ^*t*^BuOOH feeding rate of 1 mM h^−1^ approximately the same product formation rate was observed, albeit for at least 72 h. Also the formation of the undesired side products decreased considerably with lower ^*t*^BuOOH feeding rates (Fig. S19, ESI†). Both observations are most likely related to each other. At high ^*t*^BuOOH feeding rates, the peroxide availability exceeds the enzyme's epoxidation capacity resulting in oxidative inactivation of the heme prosthetic group and release of iron ions. The latter catalyse Fenton-like transformations resulting in non-selective oxidation of the *cis*-β-methylstyrene starting material and formation of the undesired side-products.

**Fig. 3:**
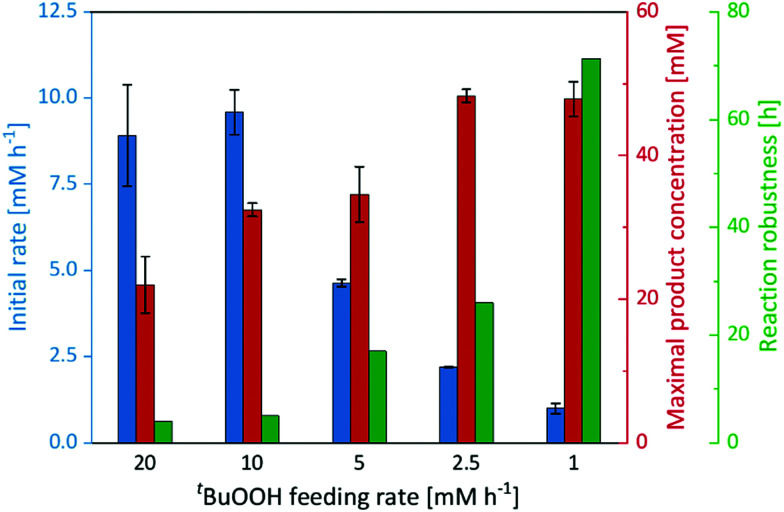
Initial rate, maximal product concentration and reaction robustness of the epoxidation of *cis*-β-methylstyrene catalysed by imm-r*Aae*UPO at different ^*t*^BuOOH feeding rates. Reaction set-up: 570 μL *cis*-β-methylstyrene, supplemented with 180 mg immobilisate and continuously fed with the indicated ^*t*^BuOOH rate. General reaction conditions: [r*Aae*UPO] = 0.5 μM, room temperature, shaking at 99 rpm with 60° angle in an overhead rotator. Data represents the average of duplicates.

In any case, lowering the ^*t*^BuOOH feeding rate not only increased the robustness of the reaction but also reduced the side-product formation. Under these conditions, 48 mM (6.4 g L^−1^) of enantiomerically pure (2*R*,3*S*)-2-methyl-3-phenyloxirane have been synthesised within 72 h. This corresponds to excellent TN of 96 000 for the biocatalyst. This catalytic performance also favourably compares to other enzyme systems such as flavin-dependent monooxygenases.^24–27^ Admittedly, the current substrate scope of the proposed immobilised r*Aae*UPO preparation is rather limited, preliminary experiments on styrene epoxidation indicate a similar catalytic potential for this substrate (Fig. S21, ESI†). Future experiments will broaden the synthetic scope of the proposed r*Aae*UPO preparation.

To obtain a first overview over the environmental impact of the reaction system established in this study, we used Sheldon's E-factor^28,29^ to estimate the wastes generated in the formation of (2*R*,3*S*)-2-methyl-3-phenyloxirane. As shown in Table 1, a total of 153.3 kg of waste was generated per kg of the desired product. 70% of the E-factor contribution stems from non-reacted starting material, which in a putative preparative-scale reaction can be recovered *via* distillation. The second-largest contributor (24%) is the enzyme preparation. The latter is mostly comprised of the alginate beads and buffer (making 99% of the total mass of the immobilised enzyme).

**Table 1:** E-Factor analysis of the epoxidation of *cis*-β-methylstyrene to (2*R*,3*S*)-2-methyl-3-phenyloxirane

Products	(2*R*,3*S*)-2-methyl-3-phenyloxirane	4.8
	^*t*^BuOH^a^	41.0
	Various side products^b^	0.9
Reactants	*cis*-β-Methylstyrene^c^	515.9
	^*t*^BuOOH^a^	0.0
Catalyst	r*Aae*UPO^d^	180.0
E-factor	SUM (waste)	737.9
	SUM (epoxide product)	4.8
	E-factor	153.3

a It is assumed that all added ^*t*^BuOOH converted into ^*t*^BuOH. The indicated ^*t*^BuOH mass includes decane which was used for dilution of ^*t*^BuOOH.

b Concentrations and masses of side products were estimated based on the GC calibration line and response factor of (2*R*,3*S*)-2-methyl-3-phenyloxirane.

c Mass of *cis*-β-methylstyrene after reaction stop is estimated based on formation of epoxide and side products.

d r*Aae*UPO includes imm-r*Aae*UPO beads.

We are convinced that further optimisation of the immobilisation protocol will improve the r*Aae*UPO loading in the alginate beads. For example using alginate-in-oil emulsions for the bead preparation will certainly improve the r*Aae*UPO loading.^30^ Also the E-factor contribution of the oxidant and its by-product ^*t*^BuOH, respectively, can be reduced significantly when co-immobilising the formate oxidase from *Aspegillus oryzae* (*Ao*FOX) to use methanol as sacrificial electron donor for the *in situ* generation of H_2_O_2_.^31–34^ Implementing this system will eliminate the ^*t*^BuOH contribution to the E-factor (8.5 kg kg^−1^) and reduce it to approx. 0.11 kg_CO2_ kg_Product_^−1^.

Overall, in this contribution we have established alginate-confined peroxygenase-CLEAs as practical enzyme preparations for the selective epoxidation of styrene derivates such as *cis*-β-methylstyrene to synthesise enantiomerically pure epoxides. In terms of catalyst efficiency (more than 90 000 catalytic cycles observed), the current system outperforms comparable reaction systems using chemical catalysts,^35^ P450 monooxygenases^36^ or other established enzymatic systems,^26,27,37^ also compared to our previous efforts using immobilised r*Aae*UPO.^9^ We are convinced that further optimisation will bring this approach to maturity and will establish an economically and environmentally feasible reaction system.

Financial support by the China Scholarship Council, the from the German Academic Exchange Service and the German Academic Scholarship Foundation as well as the European Research Council (ERC Consolidator Grant No. 648026) is gratefully acknowledged.

F. E. H. N., Y. W. and M. P. have performed the experiments and analysed the results. The study was conceptualised by M. H., M. A. and F. H. All authors contributed to the manuscript writing.

## Conflicts of interest

There are no conflicts to declare.

## Supplementary Material

CC-057-D1CC01868J-s001
