# Supplementary material for: An alginate-confined peroxygenase-CLEA for styrene epoxidation
Source: Chem Commun (Camb). 2021 May 7;57(47):5766–9. doi: 10.1039/d1cc01868j (PMC8191455; doi:10.1039/d1cc01868j)
Supplement: CC-057-D1CC01868J-s001 [file CC-057-D1CC01868J-s001.pdf]

Electronic Supplementary Material (ESI) for ChemComm.
This journal is © The Royal Society of Chemistry 2021

SUPPORTING INFORMATION

An Alginate-Confined Peroxygenase-CLEA for Styrene Epoxidation

*Friederike E. H. Nintzel,[a]† Yinqi Wu,[a]† Matteo Planchestainer,[b] Martin Held,[b] Miguel Alcalde,[c] Frank Hollmann[a]**

a. Department of Biotechnology, Delft University of Technology, van der Maasweg 9, 2629 HZ Delft.
b. Department of Biosystems Science and Engineering, ETH Zürich, Mattenstrasse 26, 4058 Basel, Switzerland.
c. Department of Biocatalysis, Institute of Catalysis and Petrochemistry (CSIC), Madrid, Spain.
† Both authors contributed equally.
*Corresponding author, E-mail: f.hollmann@tudelft.nl

Content

S1. Materials and Methods **4**

S1.1 Chemicals and materials 4

S1.2 Production of rAaeUPO 4

S1.3 Optimisation of the enzyme immobilisation process 5

S1.4 Determination of immobilisation efficiency 8

S1.5 Gas chromatography (GC) 9

S1.6 Enzymatic epoxidation reactions 11

S1.7 E-factor analysis 13

S2. Supporting Results **14**

S2.1 Production of rAaeUPO 14

S2.2 Immobilisation of rAaeUPO 14

S2.3 Epoxidation of cis-β-methylstyrene 18

S2.4 Epoxidation of styrene 23

S3. Supporting comparative table **25**

S4. References **25**

List of supporting figures

Figure S1. Reaction set-up for the enzymatic epoxidation of *cis*-β-methylstyrene. 12

Figure S2. General characterisation of r*Aae*UPO. 14

Figure S3. Selection of the optimal precipitation reagent. 14

Figure S4. Formation of r*Aae*UPO-CLEAs 15

Figure S5. Optimisation of r*Aae*UPO cross-linking 15

Figure S6. Optimisation of imm-r*Aae*UPO enzyme load 16

Figure S7. Influence of alginate concentration and counter ions on alginate confinement 16

Figure S8. Shape and size of imm-r*Aae*UPO beads of different diameters 17

Figure S9. Encapsulation of GFP visualised by fluorescence microscopy 17

Figure S10. Storage stability of imm-r*Aae*UPO 18

Figure S11. GC chromatogram of synthesised 2-methyl-3-phenyloxirane 18

Figure S12. Calibration lines for *cis*-β-methylstyrene epoxidation 18

Figure S13. Example GC chromatogram for the epoxidation of *cis*-β-methylstyrene 19

Figure S14. Product formation over time for the epoxidation of *cis*-β-methylstyrene 20

Figure S15. Visual comparison of the three reaction systems 20

Figure S16. Chromatogram of the chiral GC analysis. 21
Figure S17. GC chromatograms of the epoxidation of *cis*-β-methylstyrene 21
Figure S18. Control experiments for side product formation 22
Figure S19. Product spectrum after epoxidation of *cis*-β-methylstyrene....22
Figure S20. Example GC chromatogram for the epoxidation of styrene....23
Figure S21. Product formation during epoxidation of styrene 23
Figure S22. Epoxidation of styrene with different oxidant feed rates....24
Figure S23. Imm-r*Aae*UPO before and after the epoxidation reaction....24

List of supporting schemes

Scheme S1. Reaction equation for the epoxidation of styrene....23

List of supporting tables

Table S1. Overview on the standard method for GC analysis of the *cis*-β-methylstyrene epoxidation reactions....10
Table S2. Overview on the method for chiral GC analysis of the *cis*-β-methylstyrene epoxidation reactions....11
Table S3. Overview on the standard method for GC analysis of the styrene epoxidation reactions. 11
Table S4. Key values of the immobilisation efficiency. Data represented an average of duplicates. 17
Table S5. Summary of the results of the enzymatic epoxidation reaction with *cis*-β-methylstyrene as substrate....19
Table S6. Comparison of the catalytic performance of the proposed system with some methods reported. 25

S1. Materials and Methods

S1.1 Chemicals and materials

Alginic acid sodium salt (from brown algae, low viscosity), chitosan (low viscosity), iso-propanol, acetonitrile, ethanol, ammonium sulphate, glutaraldehyde (50% aq. solution), 2,2'-azino-bis(3-ethylbenzothiazoline-6-sulphonic acid) diammonium salt (ABTS), hydrogen peroxide (30% aq. solution), calcium chloride dihydrate, barium chloride, strontium chloride, sodium hydroxide, sodium dithionite, carbon monoxide gas, terephthalaldehyde, formaldehyde, iminodiacetic acid, bovine serum albumin (BSA), super folder green-fluorescent protein (sfGFP), bradford dye reagent, SDS loading dye, SDS protein ladder, n-heptane, ethyl acetate, triethylamine, silica paper, benzaldehyde, *cis*-β-methylstyrene, decane, tetradecane, phenylacetone, styrene, styrene oxide, phenylacetaldehyde, magnesium sulphate, methyl *tert*-butyl ether, *tert*-butyl hydroperoxide, and further commercial chemicals were purchased from Sigma-Aldrich, Fluka, Alfa-Aesar, Roche, Emsure, Messer and TCI Europe without any further purification. Ultrapure water from Milli-Q was used for all experiments.

S1.2 Production of r*Aae*UPO

S1.2.1 Enzyme expression and purification

The laboratory-evolved expression variant PaDa-1 of the peroxygenase form *Agrocybe aegerita* (r*Aae*UPO) was heterologously expressed in *Pichia pastoris* and isolated following a previously described procedure.[1]

S1.2.2 SDS-PAGE analysis

r*Aae*UPO (5 µg) in Tris/HCl buffer (20 mM, pH 7.0) was mixed with 6x SDS loading buffer. Samples were boiled for 10 min at 99 °C and centrifuged for 5 min at 13000 g. A SDS-PAGE was loaded with 5 µL r*Aae*UPO and 5 µL protein ladder. The page was run for 20 min at 80 V and for 1 h at 150 V. Proteins were detected via Coomassie staining.

S1.2.3 Bradford assay

General protein concentrations were determined by Bradford assay in 1 mL cuvettes with 20 µl protein solution in Tris/HCl buffer (20 mM, pH 7.0) and 980 µl Bradford dye reagent. Absorbance was detected at 595 nm in a UV-Vis spectrophotometer. Concentrations were quantified based on a standard curve of horseradish peroxidase.

S1.2.4 Determination of r*Aae*UPO concentration

Absorbance at 420 nm

As previously described,[2] the millimolar extinction coefficient 115 mM^{-1} cm^{-1} at 420 nm was used to determine the concentration of purified r*Aae*UPO. Herein, the UV–Vis spectrum between 350 nm and 500 nm of different dilutions of purified r*Aae*UPO in 1 mL Tris/HCl buffer (20 mM, pH 7.0) was recorded, normalised and evaluated accordingly.

CO difference spectra

To determine the amount of active r*Aae*UPO, the extinction coefficient of r*Aae*UPO from the ferrous carbon monoxide binding difference spectra (CO difference spectra) was utilised. Purified r*Aae*UPO was prepared in 1 mL Tris/HCl buffer (20 mM, pH 7.0) at different dilutions. $Na_2S_2O_4$ was added in a final concentration of 10 mM to reduce the heme–thiolate enzyme. Subsequently, the UV–Vis spectrum between 400 nm and 500 nm was recorded in a UV-Vis spectrophotometer. After saturating the enzyme solution with CO, the spectrum between 400 nm and 500 nm was measured again to record the absorbance shift which occurs due to heme-CO adduct formation in active r*Aae*UPO. The millimolar extinction coefficient 91 mM^{-1} cm^{-1} at 445 nm[3][4] was utilized to calculate the concentration and mass of active imm-r*Aae*UPO after normalisation.

S1.2.5 ABTS activity assay

Activity of r*Aae*UPO and r*Aae*UPO-CLEAs

Activity of r*Aae*UPO and r*Aae*UPO-CLEAs was determined by ABTS assay as describes earlier [5]. Measurements were performed in 1 mL of 150 mM sodium phosphate citrate buffer at pH 4.4 with 0.3 mM ABTS and 1 mM H_2O_2. Absorbance was determined every 6 sec over 60 sec at 420 nm in an UV-Vis spectrophotometer Genesys 150 from Thermo Scientific. The activities were calculated in U_{ABTS} (1 U_{ABTS} = 1 μmol_{ABTS} min^{-1}) with the millimolar extinction coefficient of ABTS ($\varepsilon_\lambda = 36.8$ mM^{-1} cm^{-1}).

Activity of imm-r*Aae*UPO

Activity of imm-r*Aae*UPO was assayed in 10 mL of 100 mM MES buffer at pH 5 with 0.3 mM ABTS and 1 mM H_2O_2. Absorbance was measured every 30 sec over 90 sec at 420 nm in UV-Vis spectrophotometer Genesys 150 from Thermo Scientific. The activities were calculated in U_{ABTS} (1 U_{ABTS} = 1 μmol_{ABTS} min^{-1}) with the millimolar extinction coefficient of ABTS ($\varepsilon_\lambda = 36.8$ mM^{-1} cm^{-1}).

Recovered activities

Recovered activities of agg-r*Aae*UPO and imm-r*Aae*UPO were calculated with use of the starting r*Aae*UPO mass in mg and expressed in percentage:

$$\text{Recovered activity of agg - r}Aae\text{UPO [\%]} = \frac{\text{agg - r}Aae\text{UPO activity } \left[U_{ABTS}\ mg^{-1}_{rAaeUPO}\right]}{\text{r}Aae\text{UPO starting activity } \left[U_{ABTS}\ mg^{-1}_{rAaeUPO}\right]} \times 100$$

$$\text{Recovered activity of imm - r}Aae\text{UPO [\%]} = \frac{\text{imm - r}Aae\text{UPO activity } \left[U_{ABTS}\ mg^{-1}_{rAaeUPO}\right]}{\text{r}Aae\text{UPO starting activity } \left[U_{ABTS}\ mg^{-1}_{rAaeUPO}\right]} \times 100$$

S1.3 Optimisation of the enzyme immobilisation process

S1.3.1 Optimised protocol for the preparation of imm-r*Aae*UPO

5 mg r*Aae*UPO (112.6 nmol) were diluted in a volume of 2.5 mL Tris/HCl buffer (20 mM, pH 7) containing 10 mg low-viscosity chitosan. The solution was precipitated with 9 times the volume iso-propanol (22.5 mL) and cross-linked with 5000 molar protein equivalents of glutaraldehyde (563 µmol).

After 2 h of incubation, the samples of r*Aae*UPO-CLEAs were centrifuged for 5 min at 4000 rpm, 4 °C and the supernatant was discarded. The pellet was washed with 5 mL Tris/HCl buffer (20 mM, pH 7) and resuspended in 500 µl Tris/HCl buffer (20 mM, pH 7). The resuspended r*Aae*UPO-CLEAs were clarified with a 40 µm sieve. A solution of 2.5% (w/v) low viscosity alginate in MilliQ water was prepared and purified by filtration through a 5 µm membrane filter. The 500 µl r*Aae*UPO-CLEAs were mixed with 9.5 mL of alginate solution.

For preparation of the imm-r*Aae*UPO alginate beads, a semi-automated encapsulator (Encapsulation Unit VAR-J30 from Nisco Engineering Inc.) was used with the following parameters: frequency 3.35 kHz, amplitude 0%, 21 hPa, 4.34 mA. The prepared alginate-enzyme suspension was transferred into a 20 mL syringe and extruded through a 150 µm diameter nozzle with 3.3 mL min^{-1} speed. The droplets were captured in 100 mL of 100 mM $CaCl_2$ hardening solution under constant agitation. After 30 min of hardening, the imm-r*Aae*UPO alginate beads were collected with a 40 µm sieve and washed with 10 mL of 5 mM $CaCl_2$ washing solution. imm-r*Aae*UPO was stored in Tris/HCl buffer (20 mM, pH 7) containing 5 mM $CaCl_2$ at 4°C.

S1.3.2 Optimisation of the precipitation reagent

To determine the optimal precipitation reagent, 1 mg r*Aae*UPO (22.5 nmol) was diluted in a volume of 500 µL Tris/HCl buffer (20 mM, pH 7). The solution was precipitated with 9 volumes of iso-propanol, acetonitrile, ethanol, or saturated ammonium sulphate solution. Afterwards, 5000 times molar excess (112.6 µmol) of glutaraldehyde was added. In addition, the same experiment was conducted without any precipitation reagent.

After 2 h of incubation, the samples of r*Aae*UPO-CLEAs were treated as described in the optimised protocol. After clarification, activity of the r*Aae*UPO-CLEAs was determined by ABTS assay and expressed as recovered activity relative to free r*Aae*UPO.

S1.3.3 Optimisation of the aggregation reagent

In order to determine the optimal cross-linking protocol, five different aggregation reagents were tested. 1 mg r*Aae*UPO (22.5 nmol) was diluted in a volume of 500 µL Tris/HCl buffer (20 mM, pH 7). The solution was precipitated with 9 volumes of iso-propanol (4.5 mL) and aggregated with glutaraldehyde, terephthalaldehyde, starch oxide/ polyaldehyde starch, formaldehyde, or iminodiacetic acid. All aggregation reagents were applied in 5000 times molar excess (112.6 µmol) compared to r*Aae*UPO. In addition, the same experiment was conducted without adding any aggregation reagent. All subsequent steps follow the optimised protocol. Activity of the resulting immobilisates was tested by ABTS assay and expressed in $U_{ABTS}\ g^{-1}_{wet\ beads}$.

The starch oxide/ polyaldehyde starch was freshly prepared as the following[6], [7]: 5 mL of a 0.7 M solution of $NaIO_4$ was brought to pH 4.0 by addition of sulfuric acid. Starch was added to reach a 1:1 molar ratio with NaIO4 (568 mg of starch). The solution was constantly stirred at 37°C – 40°C during the addition. After stirring for 3 – 4 h at 37°C – 40°C, acetone was added to the solution. The precipitate was washed with solvent and water, and subsequently dried to recover a white powder of oxidized starch.

S1.3.4 Optimisation of the co-aggregator

1 mg r*Aae*UPO (22.5 nmol) was diluted in a volume of 500 µL Tris/HCl buffer (20 mM, pH 7). Bovine serum albumin (BSA) and chitosan were tested separately and in combination as co-aggregators. The compounds were used in double the quantity (2 mg) of r*Aae*UPO and added to the prepared solution. In addition, the same experiment was performed without addition of a co-aggregator. The mixture was precipitated with 9 times the volume iso-propanol (4.5 mL) and aggregated with 5000 molar protein equivalents of glutaraldehyde (112,6 µmol/ 22.5 µl of a 50% stock). All subsequent steps follow the optimised protocol. Activity of the resulting immobilisates was tested by ABTS assay and expressed in U_{ABTS} $g^{-1}{}_{wet\ beads}$.

S1.3.5 Optimisation of the glutaraldehyde concentration

1 mg r*Aae*UPO (22.5 nmol) and 2 mg chitosan were diluted in a volume of 500 µL Tris/HCl buffer (20 mM, pH 7). The mixture was precipitated with 9 times the volume iso-propanol (4.5 mL). To test different concentrations of the aggregation reagent glutaraldehyde, 500 molar r*Aae*UPO equivalents, 5000 molar r*Aae*UPO equivalents, and 50000 molar r*Aae*UPO equivalents were used for aggregation of the protein-chitosan mix. All subsequent steps follow the optimised protocol. Activity of the resulting immobilisates was tested by ABTS assay and expressed in U_{ABTS} $g^{-1}{}_{wet\ beads}$.

S1.3.6 Optimisation of the catalyst loading

In order to determine the optimal catalyst loading, the immobilisation of 1 mg, 2 mg, 5 mg and 10 mg r*Aae*UPO was analysed. 1 mg / 2 mg / 5 mg / 10 mg r*Aae*UPO and 2 mg / 4 mg / 10 mg / 20 mg chitosan were diluted in a volume of 500 µL / 1 mL / 2.5 mL / 5 mL Tris-HCl buffer (20 mM, pH 7). The mixture was precipitated with 9 times the volume iso-propanol and aggregated with 5000 molar protein equivalents of glutaraldehyde. All subsequent steps follow the optimised protocol. Activity of the resulting immobilisates was tested by ABTS assay and expressed in U_{ABTS} $g^{-1}{}_{wet\ beads}$. Moreover, recovered activity relative to free r*Aae*UPO was determined.

S1.3.7 Optimisation of the alginate concentration

Resuspended r*Aae*UPO-CLEAs were prepared as given in the optimised protocol und clarified with a 40 µm sieve. Solutions of 2.5%, 3%, 3.5%, and 4% low viscosity alginate in MilliQ water were prepared and purified by filtration through a 5 µm membrane filter. The 500 µl r*Aae*UPO-CLEAs were mixed with 9.5 mL of the alginate solutions. All subsequent steps follow the optimised protocol. Activity of the resulting immobilisates was tested by ABTS assay and expressed in U_{ABTS} $g^{-1}{}_{wet\ beads}$.

S1.3.8 Optimisation of the counterions

An alginate-enzyme suspension was prepared as described in the optimised protocol. It was transferred into a 20 mL syringe and extruded through a 150 µm diameter nozzle with 3.3 mL min^{-1} speed using a semi-automated encapsulator set-up as outlined in the optimised protocol. In order to test the effect of counterions used for hardening of the alginate beads, 50 mM $CaCl_2$, 100 mM $CaCl_2$, 200mM $CaCl_2$, 100mM $BaCl_2$, and 100 mM $SrCl_2$ were examined as hardening solutions. The alginate-enzyme droplets were captured in 100 mL of these solutions under constant agitation. After 30 min of hardening, the imm-r*Aae*UPO alginate beads were collected with a 40 µm sieve and washed with 10 mL of 5 mM $CaCl_2$, 5mM $BaCl_2$, or 5 mM $SrCl_2$ washing

solution. imm-r*Aae*UPO was stored in 20 mM Tris/HCl buffer (pH 7) containing 5 mM $CaCl_2$, 5mM $BaCl_2$, or 5 mM $SrCl_2$ at 4°C. Activity of the resulting immobilisates was tested by ABTS assay and expressed in U_{ABTS} $g^{-1}{}_{wet\ beads}$.

S1.3.9 Optimisation of the alginate bead diameter

The immobilisation was performed as described in the optimised protocol. Next to using a 150 µm diameter nozzle in the semi-automated encapsulation unit, nozzles of 50 µm, 100 µm, 200 µm, and 400 µm diameter were utilized to examine the effect of beads size on the immobilisation efficiency.

For manual preparation of imm-r*Aae*UPO, an alginate-enzyme suspension was prepared as described in the optimised protocol and was transferred into a 20 mL syringe connected to a 0.5 mm x 16 mm cannula. With use of a syringe pump, the alginate-enzyme suspension was extruded at a constant speed of 3.3 mL min^{-1}. The droplets were captured in 100 mL of 100 mM $CaCl_2$ hardening solution under constant agitation. Hardening, washing, and storage were performed as in the optimised protocol. Activity of all resulting immobilisates was tested by ABTS assay and expressed in U_{ABTS} $g^{-1}{}_{wet\ beads}$.

S1.4 Determination of immobilisation efficiency

S1.4.1 Determination of the immobilisation yield

The immobilisation yield was obtained by using two different approaches. First, the general r*Aae*UPO concentration within the immobilisates was quantified with use of the millimolar extinction coefficient at 420 nm. Moreover, the concentration of active heme-sites inside imm-r*Aae*UPO was determined by CO difference spectra.

Herein, 200 mg of dry imm-r*Aae*UPO beads were dissolved in 5 mL of 150 mM sodium phosphate citrate buffer at pH 4.4 and incubated for 30 min. The solution was neutralised with NaOH and the concentration of r*Aae*UPO inside the solution was determined with use of the millimolar extinction coefficient at 420 nm. Afterwards, the solution was concentrated to a volume of 1 mL using a centrifugal filter unit Ulra-4 with 10 kDa molecular weight cut-off from Amicon. The r*Aae*UPO concentration inside the resulting solution was quantified by CO difference spectra. The immobilization yield was determined relative to the starting mass of r*Aae*UPO of 5 mg:

$$\text{Immobilisation yield [\%]} = \frac{\text{Mass of imm - r}\mathit{Aae}\text{UPO [mg]}}{\text{Starting mass of r}\mathit{Aae}\text{UPO [mg]}} \times 100$$

S1.4.2 Analysis of storage stability

A sample of imm-r*Aae*UPO alginate beads as well as r*Aae*UPO enzyme stock was stored at 4°C and at room temperature and activity over time was tracked by ABTS assay. The stability was calculated in percentage:

$$\mathit{Stability}\ [\%] = \frac{\text{Activity at } \mathit{sample} \text{ timepoint [U mg}^{-1}_{\text{r}\mathit{Aae}\text{UPO}}]}{\text{Starting activity [U mg}^{-1}_{\text{r}\mathit{Aae}\text{UPO}}]}$$

S1.4.3 Light and fluorescence microscopy

Samples analysed by light microscopy were prepared following the optimized immobilisation protocol. Samples for fluorescence microscopy were prepared respectively with the following deviations from the protocol: 1 mg sfGFP[8] was diluted in a volume of 300 µl Tris/HCl buffer (20 mM, pH 7). The suspension was clarified with a 40 µm sieve. A solution of 2.5% low viscosity alginate in MilliQ water was prepared and purified by filtration through a 5 µm membrane filter. The 300 µl GFP was mixed with 4.7 mL of alginate solution.

Light microscopy pictures were taken with use of the white light channel of Microscope Axio Observer A1 from ZEISS. Fluorescence microscopy pictures were taken as overlay of the while light channel and GFP channel. Size measurement was performed with the inbuilt ZEISS software.

S1.5 Gas chromatography (GC)

S1.5.1 Chemical synthesis of GC standard 2-methyl-3-phenyloxirane

In order to serve as standard chemical for analytics, racemic 2-methyl-3-phenyloxirane was synthesised as previously described[9]. After synthesis, qualitative analysis of the product purity was performed with Thin-Layer-Chromatography (TLC). Herein, silica paper was used as solid phase. A 98:2 mixture of n-heptane and ethyl acetate including 1% triethylamine for basification of the silica paper was used as mobile phase.

Purification of the product mixture was undertaken with an automated Reveleris X2 flash purification system. A 4 g Reveleris silica column was used as solid phase and a 98:2 mixture of n-heptane and ethyl acetate including 1% triethylamine was used as single mobile phase at 15 mL min^{-1} flow rate. Product fractions were collected within the first 3 minutes and analysed by TLC and gas chromatography. Fractions with high purity were pooled and remaining solvent was evaporated using a rotary vacuum evaporator.

S1.5.2 GC for *cis*-β-methylstyrene epoxidation

Product mixtures of *cis*-β-methylstyrene epoxidation were analysed using a Shimadzu GC-2014 gas chromatograph with a flame ionization detector and a CP Sil 5 CB column (dimensions: 50.0 m x 0.53 mm x 1.00 µm nominal). Carrier gas: Nitrogen; Column flow: 20 mL/min. The utilised temperature profile and retention times of all analysed compounds are given in the following (Table S1).

Concentrations of 2-methyl-3-phenyloxirane, benzaldehyde and phenylacetone were quantified based on calibration lines and with use of an internal standard tetradecane. Calibration lines were set up in duplicates by preparing respective concentrations including 100 mM tetradecane in styrene as solvent. 5 µL of this mixture was diluted in 1 mL of methyl *tert*-butyl ether and dried with $MgSO_4$ before GC analysis. Commercially available standard chemicals were used for the calibration lines of benzaldehyde and phenylacetone. For 2-methyl-3-phenyloxirane, a self-synthesised standard was used. In all cases, standard purities based on peak area ratios were taken into account by only using the peak area at expected retention times for the calibration lines.

Table S1. Overview on the standard method for GC analysis of the *cis*-β-methylstyrene epoxidation reactions.

Rate (°C min^{-1})	Temp (°C)	Hold (min)
-	70	5
30	80	3
30	106	4
30	160	2
30	340	1

Compound	Retention time (min)
Benzaldehyde (by-product)	6.32
cis-β-Methylstyrene (substrate)	7.37
Decane (solvent of ^{t}BuOOH)	7.92
By-product A	8.71
2-methyl-3-phenyloxirane (product)	10.51
By-product B	10.82
Phenylacetone (by-product)	11.15
By-product C	14.16
By-product D	14.82
By-product E	15.23
By-product F	15.46
Tetradecane (internal standard)	16.38

S1.5.3 Chiral GC for *cis*-β-methylstyrene epoxidation

To determine enantiomeric excess (*ee*), product mixtures of *cis*-β-methylstyrene epoxidation were analysed as previously described[9] by chiral GC (Shimadzu GC-2010) with a flame ionization detector and a Lipodex E 1b (Macherey-Nagel) column (dimensions: 50.0 m × 0.25 mm × 0.25 µm). Carrier gas: Helium; Column flow: 2.16 mL/min; Split ratio: 100; Linear velocity: 38 cm/s. The utilised temperature profile and retention times of all analysed compounds are given in the following (Table S2). Enantiomers were quantified by peak area integration and the *ee* was calculated as:

$$ee = \frac{peak\ area\ (R) - peak\ area\ (S)}{peak\ area\ (R) + peak\ area\ (S)}$$

Table S2. Overview on the method for chiral GC analysis of the *cis*-β-methylstyrene epoxidation reactions.

Rate (°C min^{-1})	Temp (°C)	Hold (min)
-	100	15
20	220	1

Compound	Retention time (min)
Styrene (solvent of epoxide)	4.00
cis-β-Methylstyrene (substrate)	5.20
(2*R*,3*S*)-2-methyl-3-phenyloxirane (product)	10.31
(2*S*,3*R*)-2-methyl-3-phenyloxirane (product)	13.24
Tetradecane (internal standard)	18.21

S1.5.4 GC for styrene epoxidation

Product mixtures of styrene epoxidation were analysed by chiral GC (Gas chromatography GC System 6890 Series from Agilent) with a flame ionization detector and a CP-Chiasil-Dex-CB column from Agilent (dimensions: 25.0 m x 250 µm x 0.25 µm nominal). Helium served as carrier gas and the injection volume was 5 µL. Product formation was determined as GC conversion, obtained with peak area integrations of the product mixture. The utilised temperature profile and retention times of all analysed compounds are given in the following (Table S3).

Table S3. Overview on the standard method for GC analysis of the styrene epoxidation reactions.

Rate (°C min^{-1})	Temp (°C)	Hold (min)
-	70	1
20	200	1

Compound	Retention time (min)
Styrene (substrate)	3.10
Styrene oxide and phenylacetaldehyde (products)	approx. 4.83
Tetradecane (internal standard)	6.21

S1.6 Enzymatic epoxidation reactions

S1.6.1 Epoxidation of *cis*-β-methylstyrene

The reaction was performed in small GC vials at room temperature, shaking at 99 rpm with 60° angle in an overhead rotator (Figure S1). *tert*-Butyl hydroperoxide (^{t}BuOOH) was fed continuously with use of a syringe pump and 1 mL syringes which were connected with tubes to the reaction vials.

Before start of the reaction, *cis*-β-methylstyrene was supplemented with 100 mM tetradecane as internal standard and saturated with TRIS/HCl buffer (20 mM, pH 7). Moreover, a 5 M stock of ^{t}BuOOH in decane was supplemented with 100 mM internal standard tetradecane. If further dilution of ^{t}BuOOH was required, decane was used as solvent.

A GC vial was prepared with 0.5 µM r*Aae*UPO. For the case of imm-r*Aae*UPO, 180 mg of carefully dried immobilisates were utilised. For the case of free r*Aae*UPO in a two liquid phase system, 0.5 µM enzyme was prepared in an aqueous phase of 180 µL TRIS/HCl buffer (20 mM, pH 7). For the case of free r*Aae*UPO in a micro-aqueous system, 0.5 µM enzyme was prepared in an aqueous phase of 6.6 µL TRIS/HCl buffer (20 mM, pH 7).

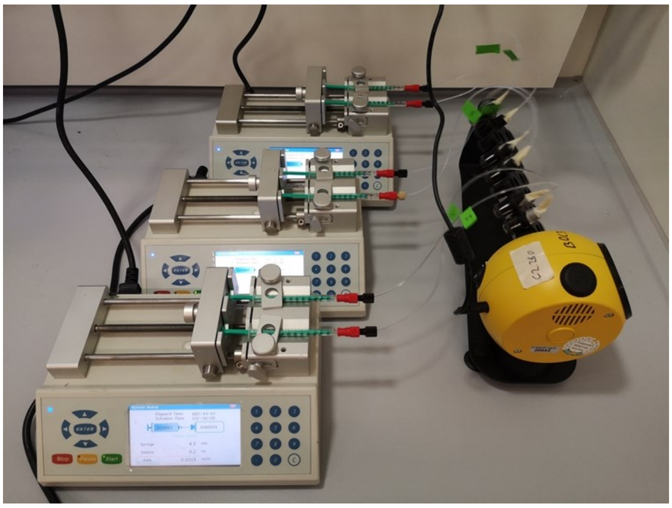

Figure S1. Reaction set-up for the enzymatic epoxidation of *cis*-β-methylstyrene. On the left, syringe pumps for continuous ^{t}BuOOH supply. Syringes are connected to tubes which lead to the reaction mixture inside GC vials on the right. An overhead rotator is used for shaking of the reaction at 99 rpm, 60 ° at room temperature.

To start the reaction, the prepared *cis*-β-methylstyrene was added to the GC vial to reach a final volume of 750 µL. Subsequently, the vials were connected to the continuous ^{t}BuOOH feed. The utilised feed rate is indicated in the caption of the respective figures. 5 µL samples were taken at indicated time points, diluted in 1 mL methyl *tert*-butyl ether, and dried with $MgSO_4$. Product mixtures were analysed by gas chromatography. Turnover frequencies (TOF) and turnover numbers (TN) were calculated as the following:

$$\text{TOF}\ [\text{s}^{-1}] = \frac{\text{Concentration } of\ product\ [\text{mM}]}{\text{Concentration of enzyme [mM]} \times \text{time [s]}} \qquad \text{TN} = \frac{\text{Concentration of product [mM]}}{\text{Concentration of enzyme [mM]}}$$

S1.6.2 Epoxidation of styrene

Continuous ^{t}BuOOH feed

The same set-up as for the epoxidation of *cis*-β-methylstyrene was used. Styrene was prepared with 2% internal standard tetradecane and saturated with TRIS/HCl buffer (20 mM, pH 7). If required, dilution of ^{t}BuOOH was performed with decane as solvent. A GC vial was prepared with 0.8 µM carefully dried imm-r*Aae*UPO beads. 1 mL of styrene was added into the vial. The reaction was started with a pulse of ^{t}BuOOH of 1 mM final concentration. Subsequently, the vials were connected to the continuous ^{t}BuOOH feed. The feed rate during the biotransformation is indicated in the caption of the respective figures. Samples were taken at indicated time points, diluted in 1 mL methyl *tert*-butyl ether, dried with $MgSO_4$, and analysed by gas chromatography.

^{t}BuOOH pulse feeding

Styrene and ^{t}BuOOH were prepared as for the reactions with continuous feed. 0.2 µM r*Aae*UPO were provided in a GC vial as carefully dried imm-r*Aae*UPO or in a micro-aqueous system with 2 µL phase of TRIS/HCl buffer (20 mM, pH 7). 500 µl of styrene were added and the reaction was started with the addition of ^{t}BuOOH in pulses of 1.25 mM final concentration every 15 min. Samples were taken at indicated time points, diluted in 1 mL methyl *tert*-butyl ether, dried with $MgSO_4$, and analysed by gas chromatography.

S1.7 E-factor analysis

The E-factors were calculated according to literature[10]. All waste that was produced during a reaction was taken into account, including water:

$$E - factor = \frac{\sum m_{wastes}\,[kg]}{m_{product}\,[kg]}$$

S2. Supporting Results

S2.1 Production of r*Aae*UPO

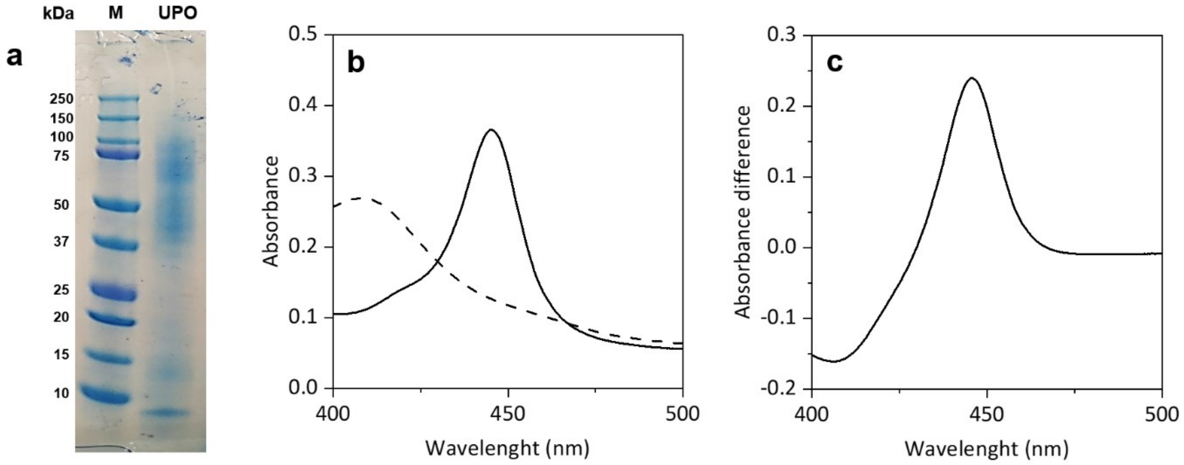

Figure S2. General characterisation of r*Aae*UPO. (a) SDS-PAGE analysis of purified r*Aae*UPO. M = molecular weight marker; UPO = r*Aae*UPO. Expected molecular weight of r*Aae*UPO is 44.4 kDa.[1] [11] (b) Spectra of purified r*Aae*UPO after reduction with $Na_2S_2O_4$ (dashed line) and after incubation with carbon monoxide (solid line). (c) CO difference spectra of purified r*Aae*UPO.

S2.2 Immobilisation of r*Aae*UPO

S2.2.1 Optimisation of CLEA formation

Precipitation

Iso-propanol was selected as optimal precipitation reagent due to a high recovered activity, its easy handling, and its low hazardousness.

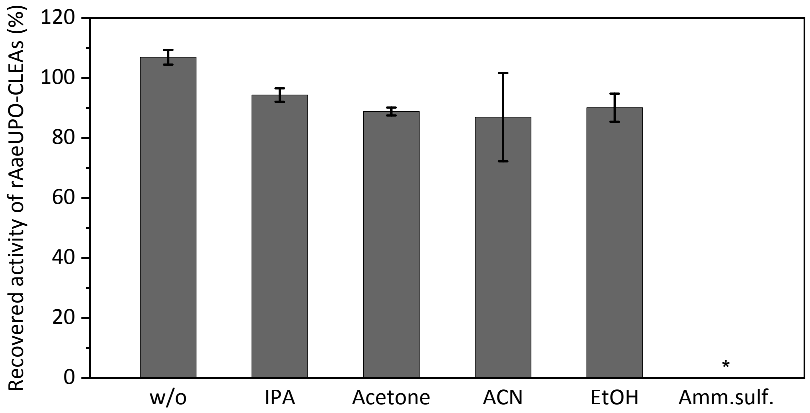

Figure S3. Selection of the optimal precipitation reagent, used for precipitation prior to CLEA formation. Recovered activity of r*Aae*UPO-CLEAs after precipitation with different precipitation reagents was determined relative to free r*Aae*UPO by ABTS-activity assay in aqueous environment. w/o = no precipitation reagent; IPA = iso-propanol; ACN = acetonitrile; EtOH = ethanol; Amm.sulf. = saturated ammonium sulphate solution. At conditions, indicated with asterisk (*), no enzyme precipitation was observed. Data represents an average of duplicates.

Cross-linking

5000 molar equivalents of glutaraldehyde were chosen for cross-linking and chitosan was selected as co-aggregator since respective immobilisates yielded highest ABTS oxidation activities.

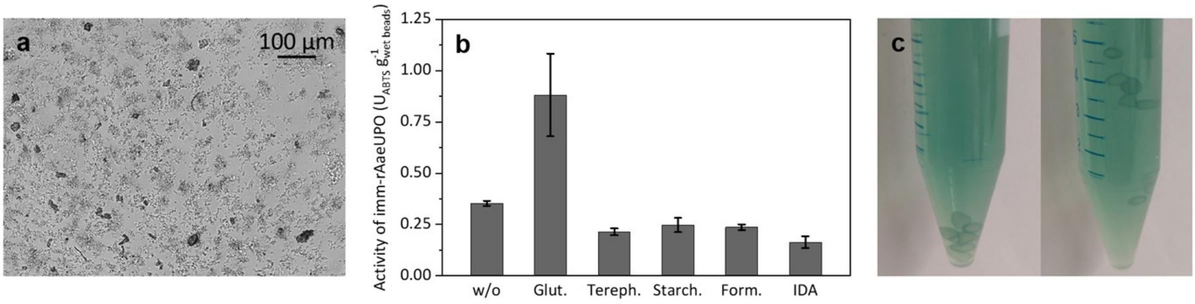

Figure S4. Formation of r*Aae*UPO-CLEAs. (a) Resuspended and clarified r*Aae*UPO CLEAs, prepared with glutaraldehyde. (b) Absolute activity of imm-r*Aae*UPO prepared with different aggregation reagents, determined by ABTS-activity assay in aqueous environment. Glut. = glutaraldehyde; Tereph. = Terephthalaldehyde; Starch. = starch oxide/ polyaldehyde starch; Form. = formaldehyde; IDA = iminodiacetic acid; w/o = no aggregation reagent as control. Data represents an average of duplicates. (c) ABTS oxidation product accumulates at the outer layer of the imm-r*Aae*UPO beads. Here, beads with 2 mm diameter were used.

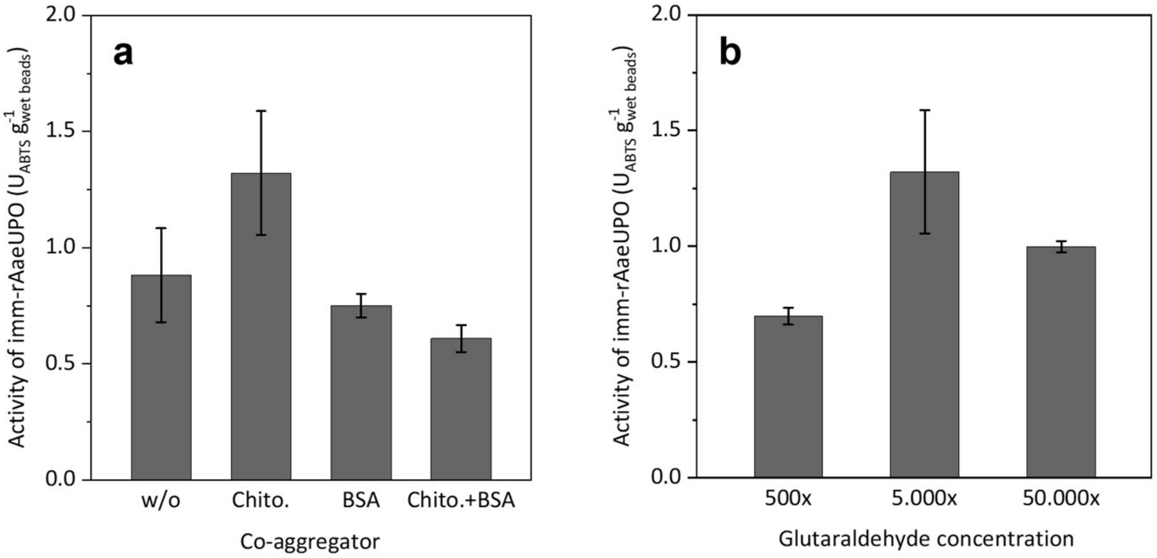

Figure S5. Optimisation of r*Aae*UPO cross-linking. Activity of imm-r*Aae*UPO at different immobilisation conditions, determined by ABTS-activity assay in aqueous environment. Data represents an average of duplicates. (a) Selection of the optimal co-aggregator. w/o = no co-aggregator as control; Chito. = chitosan. (B) Optimisation of glutaraldehyde concentration. 'x' indicates molar equivalents of enzyme.

S2.2.2 Optimisation of alginate confinement

Catalyst loading

An enzyme loading of 0.5 $mg_{Protein}$ g^{-1}_{beads} was used in the optimised immobilisation protocol.

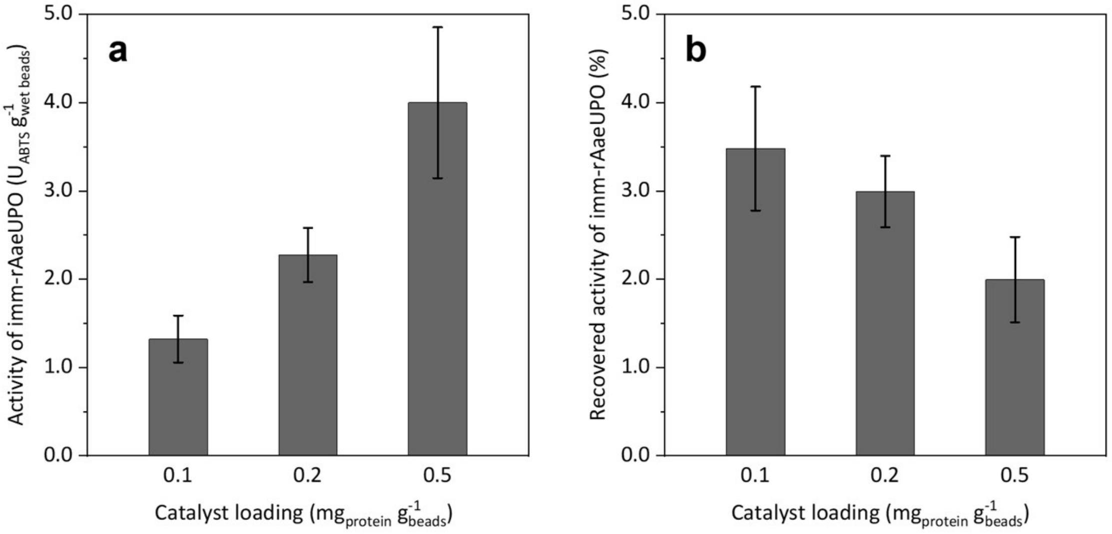

Figure S6. Optimisation of imm-r*Aae*UPO enzyme loading. Activities of imm-r*Aae*UPO at different loadings of r*Aae*UPO were determined by ABTS-activity assay in aqueous environment. Data represents an average of duplicates. Catalyst loading of 1 $mg_{Protein}$ g^{-1}_{beads} did not yield functional catalyst beads. (a) Absolute activity of imm-r*Aae*UPO. (b) Recovered activity of imm-r*Aae*UPO, determined relative to free r*Aae*UPO.

Alginate concentration and counter ions

In the final immobilisation protocol, 2.5% alginate was utilised and 100 mM $CaCl_2$ was used for hardening of the immobilisates.

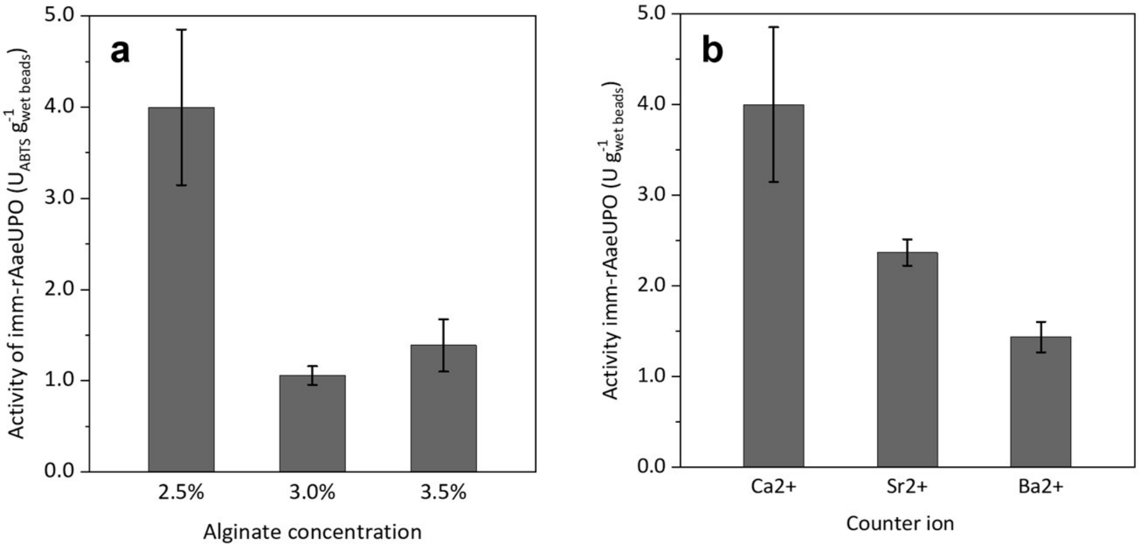

Figure S7. Influence of alginate concentration and counter ions on alginate confinement. Activity of imm-r*Aae*UPO at different immobilisation conditions, determined by ABTS-activity assay in aqueous environment. Data represents an average of duplicates. (a) Selection of the optimal alginate concentration. 4% alginate concentration did not yield functional catalyst beads. (b) Determination of the most suited counter ion.

Diameter of the immobilisate

In the final immobilisation protocol, imm-r*Aae*UPO beads of 440 µm diameter were prepared as they were most homogeneous in size and shape. Moreover, an even distribution of protein inside the alginate beads was observed after encapsulating non-cross linked GFP.

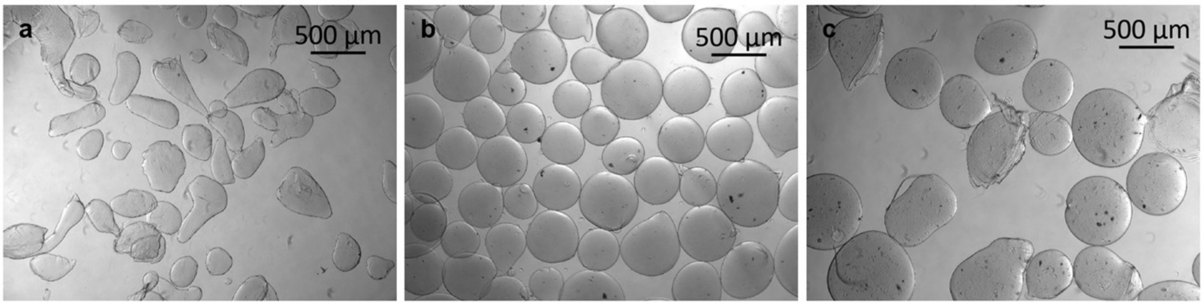

Figure S8. Shape and size of imm-r*Aae*UPO beads of different diameters. Since a semi-automated encapsulation unit was used for the preparation of the enzyme alginate beads, the beads size could be controlled by the choice of the nozzle diameter. (a) 100 µm nozzle diameter yielded 290 µm diameter beads; (b) 150 µm nozzle diameter yielded 440 µm diameter beads; (c) 200 µm nozzle diameter yielded 570 µm diameter beads. For 50 µm and 400 µm nozzle diameter, the formation of alginate beads was not possible.

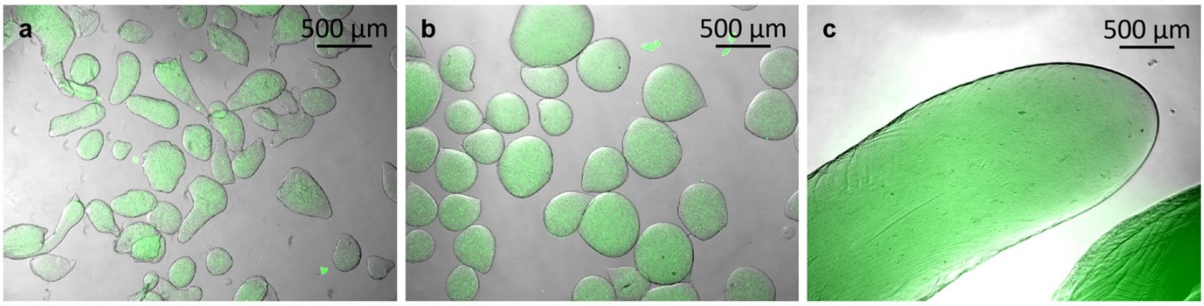

Figure S9. Encapsulation of GFP visualised by fluorescence microscopy. Free GFP was confined in calcium alginate beads of different diameters. (a) 290 µm diameter beads; (b) 440 µm diameter beads; (c) 2 mm diameter beads.

S2.2.3 Immobilisation efficiency

Table S4. Key values of the immobilisation efficiency. Data represented an average of duplicates.

	**Key value [%]**
**Immobilisation yield of imm-r*Aae*UPO[a]**	18.5 ± 4.0
**Immobilisation yield of imm-r*Aae*UPO[b]**	18.7 ± 3.1
**Relative specific activity of imm-r*Aae*UPO[c]**	11.4 ± 2.2
**Recovered activity of r*Aae*UPO-CLEAs**	47.8 ± 3.8
**Recovered activity of imm-r*Aae*UPO**	2.1 ± 0.4

[a] Yield determined leveraging the absorbance peak of r*Aae*UPO at 420nm for the total concentration of r*Aae*UPO.
[b] Yield determined as active heme sites inside imm-r*Aae*UPO by CO-differential spectra.
[c] Specific activity for ABTS oxidation in aqueous environment was 44.4 U_{ABTS} $mg^{-1}_{protein}$. Here, expressed relative to free r*Aae*UPO.

S2.2.4 Storage stability of imm-r*Aae*UPO

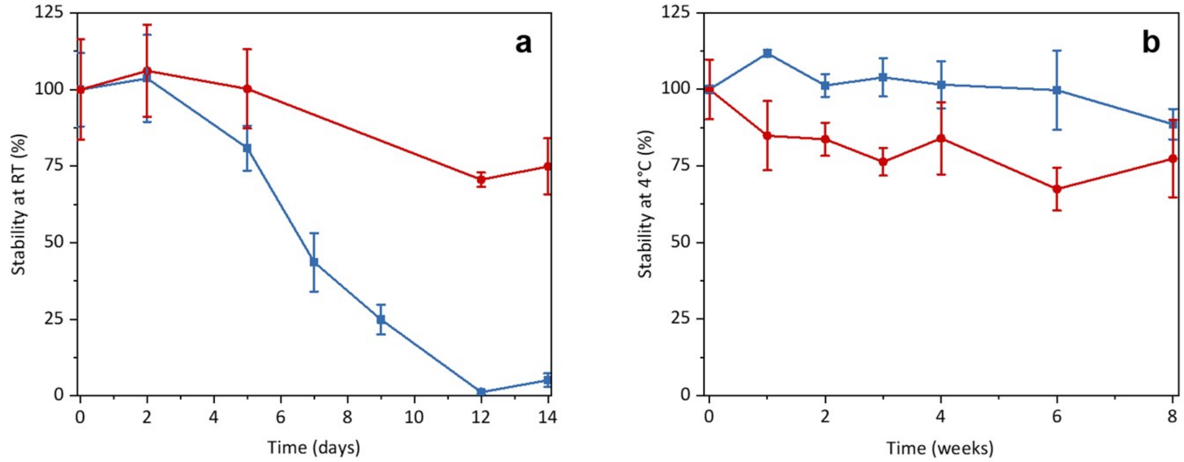

Figure S10. Storage stability of imm-r*Aae*UPO. The stability during storage of immobilisate (red circles) was analysed in comparison to the storage stability of free r*Aae*UPO (blue squares). Data was obtained by ABTS-activity assay in aqueous environment and represents an average of triplicates. (a) Storge at room temperature for 14 days. (b) Storage at 4 °C for 8 weeks.

S2.3 Epoxidation of *cis*-β-methylstyrene

S2.3.1 Synthesis of epoxide standard and preparation of GC calibration lines

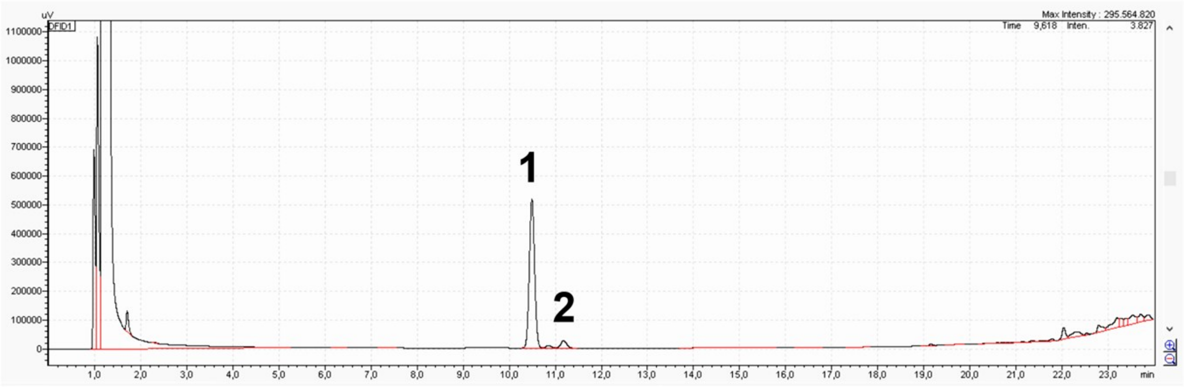

Figure S11. GC chromatogram of the synthesised standard chemical 2-methyl-3-phenyloxirane (racemic) after purification. 1 = 2-methyl-3-phenyloxirane; 2 = phenylacetone. Final purity of about 90% was estimated based on peak area ratio.

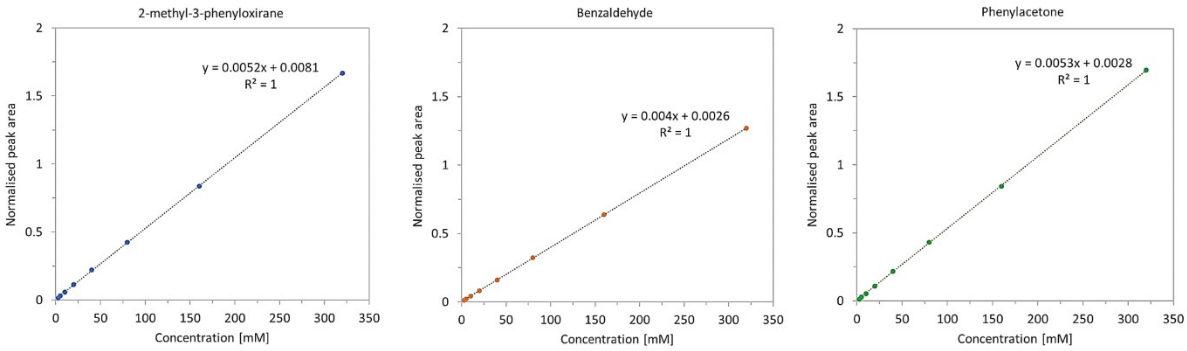

Figure S12. Calibration lines for the quantification of 2-methyl-3-phenyloxirane, benzaldehyde and phenylacetone by gas chromatography, obtained based on 100 mM internal standard tetradecane. Data represented an average of duplicates.

S2.3.2 Additional information for the enzymatic epoxidation of *cis*-β-methylstyrene

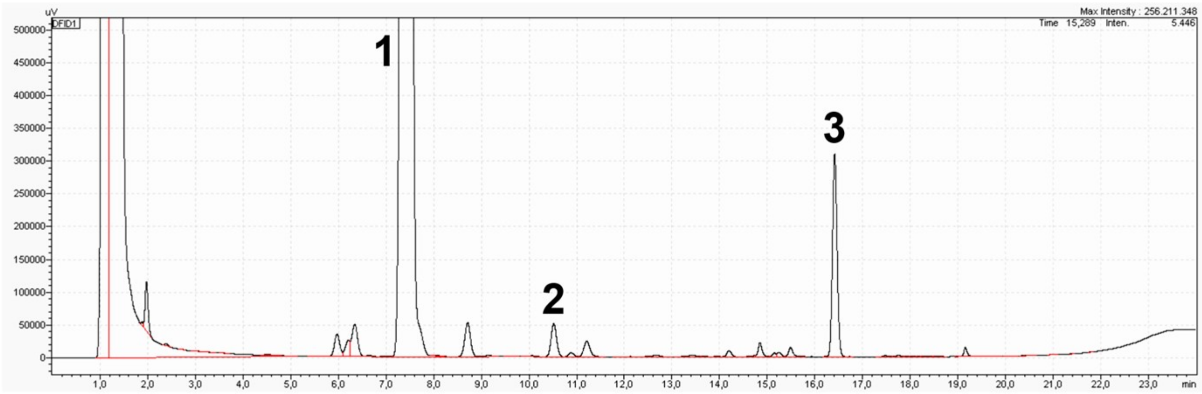

Figure S13. Example GC chromatogram for the epoxidation of *cis*-β-methylstyrene. Here, the reaction catalysed by imm-r*Aae*UPO with 10 mM h^{-1} *t*BuOOH feed is shown. 1 = *cis*-β-Methylstyrene; 2 = (2*R*,3*S*)-2-methyl-3-phenyloxirane; 3 = Tetradecane (internal standard).

Table S5. Summary of the results of the enzymatic epoxidation reaction with *cis*-β-methylstyrene as substrate.

	*t*BuOOH feed [mM h^{-1}]	Reaction robustness[a] [h]	Final epoxide concentration[b] [mM]	Initial TOF[c] [s^{-1}]	Overall TN[b]
**Free r*Aae*UPO in two-liquid phase system**	10	22	9.3 ± 0.3	1.3 ± 0.3	19,000
**Free r*Aae*UPO in micro-aqueous system**	10	24	7.6 ± 0.3	1.5 ± 0.3	15,000
**imm-r*Aae*UPO**	20	4	22.0 ± 3.9	5.0 ± 0.1	44,000
**imm-r*Aae*UPO**	10	5	32.4 ± 0.9	5.3 ± 0.1	65,000
**imm-r*Aae*UPO**	5	22	34.6 ± 3.8	2.6 ± 0.6	69,000
**imm-r*Aae*UPO**	2.5	26	48.3 ± 0.9	1.2 ± 0.4	97,000
**imm-r*Aae*UPO**	1	72	47.8 ± 2.4	0.7 ± 0.3	96,000

[a] Reaction robustness is defined as timepoint when no product formation was observed anymore.
[b] Final epoxide concentration and turnover number (TN = $mol_{Product} \times mol_{rAaeUPO}^{-1}$) were quantified at reaction stop.
[c] Turnover frequency (TOF = $mol_{Product} \times mol_{rAaeUPO}^{-1} \times s^{-1}$).
General reaction conditions: [r*Aae*UPO] = 0.5 µM as imm-r*Aae*UPO (180 mg immobilisate in 570 µL *cis*-β-methylstyrene), free r*Aae*UPO in two-liquid phase system (180 µL TRIS/HCl buffer (20 mM, pH 7), 570 µL *cis*-β-methylstyrene), and free r*Aae*UPO in micro-aqueous system (6.6 µL TRIS/HCl buffer (20 mM, pH 7), 743.4 µL *cis*-β-methylstyrene); Room temperature; shaking at 99 rpm with 60° angle in an overhead rotator. Data represented an average of duplicates.

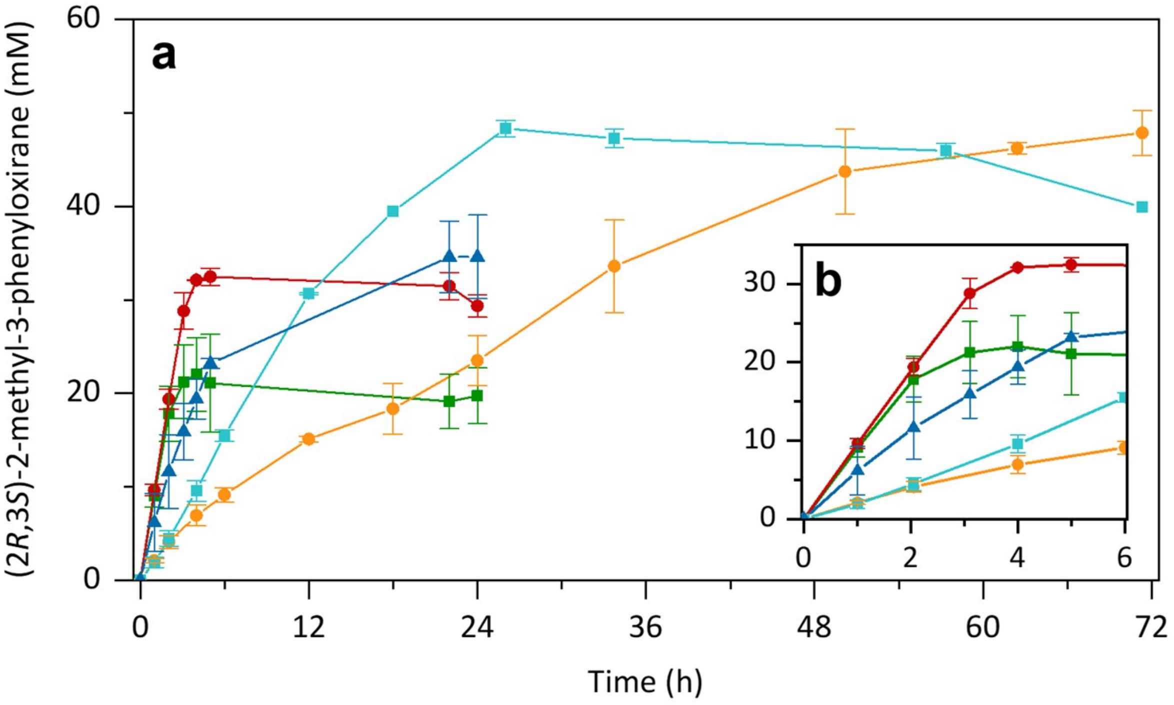

Figure S14. Product formation over time for the epoxidation of *cis*-β-methylstyrene with different *t*BuOOH feed rates. 180 mg imm-r*Aae*UPO were assayed in 570 µL neat substrate, corresponding to an enzyme concentration of 0.5 µM in the whole reaction volume. *t*BuOOH was continuously fed at rates of 20 mM h^{-1} (green square), 10 mM h^{-1} (red circle), 5 mM h^{-1} (dark blue triangle), 2.5 mM h^{-1} (light blue square), and 1 mM h^{-1} (orange circle). Data represents the average of duplicates. (a) Time-course over 72h and (b) close-up of the first 6h.

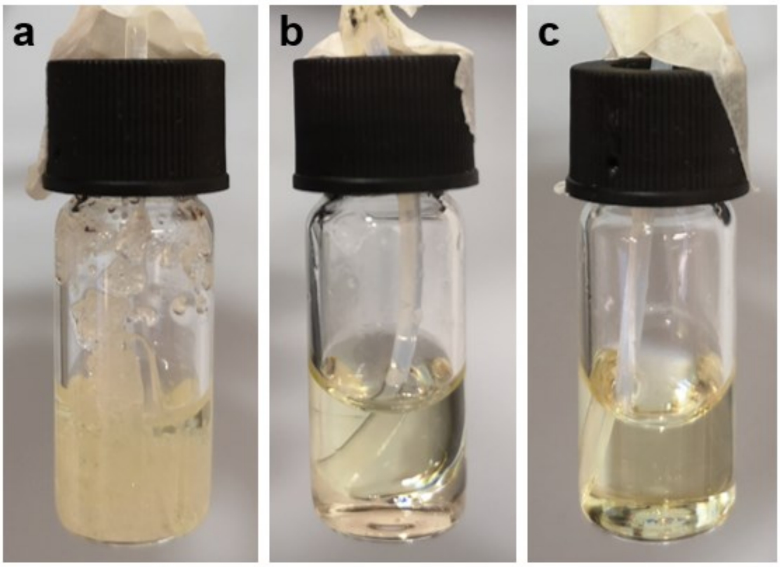

Figure S15. Visual comparison of the three reaction systems. (a) 180 mg imm-r*Aae*UPO immobilisates dispersed in 570 µL *cis*-β-methylstyrene. (b) Two liquid phase system with free r*Aae*UPO in 180 µL buffer (bottom phase) and 570 µL *cis*-β-methylstyrene (top phase). (c) Micro-aqueous system with free r*Aae*UPO in 6.6 µL buffer (small droplets on the bottom of the flask) and 743.4 µL *cis*-β-methylstyrene.

S2.3.3 Enantiomeric-excess

While the chemically synthesised epoxide standard was found to be almost fully racemic, the product mix synthesised by imm-r*Aae*UPO displayed an *ee* value of > 99 % with (2*R*,3*S*)-2-methyl-3-phenyloxirane as only product.

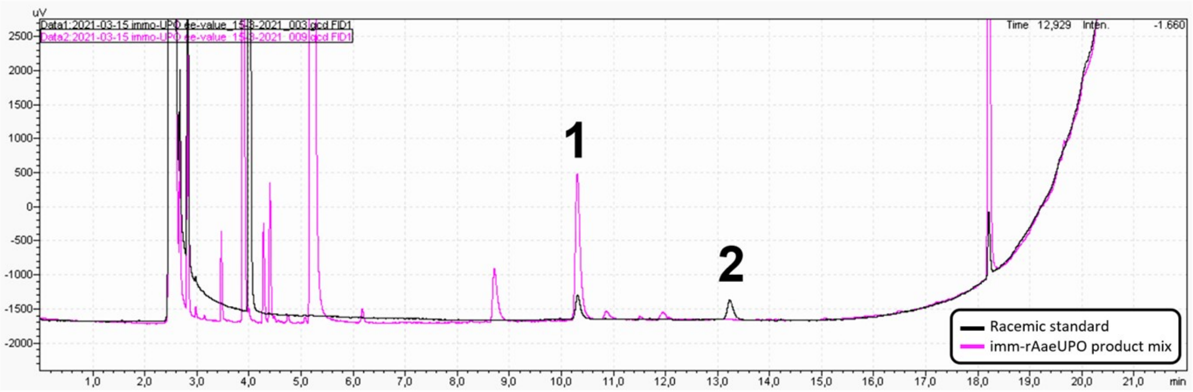

Figure S16. Chromatogram of the chiral GC analysis of an imm-r*Aae*UPO product mixture. The epoxidation of *cis*-β-methylstyrene with 1 mM h^{-1} ^{t}BuOOH feed was analysed after 72 h. Black = Chemically synthesised racemic standard; Pink = imm-r*Aae*UPO product mix; 1 = (2*R*,3*S*)-2-methyl-3-phenyloxirane; 2 = (2*S*,3*R*)-2-methyl-3-phenyloxirane.

S2.3.4 Side product formation

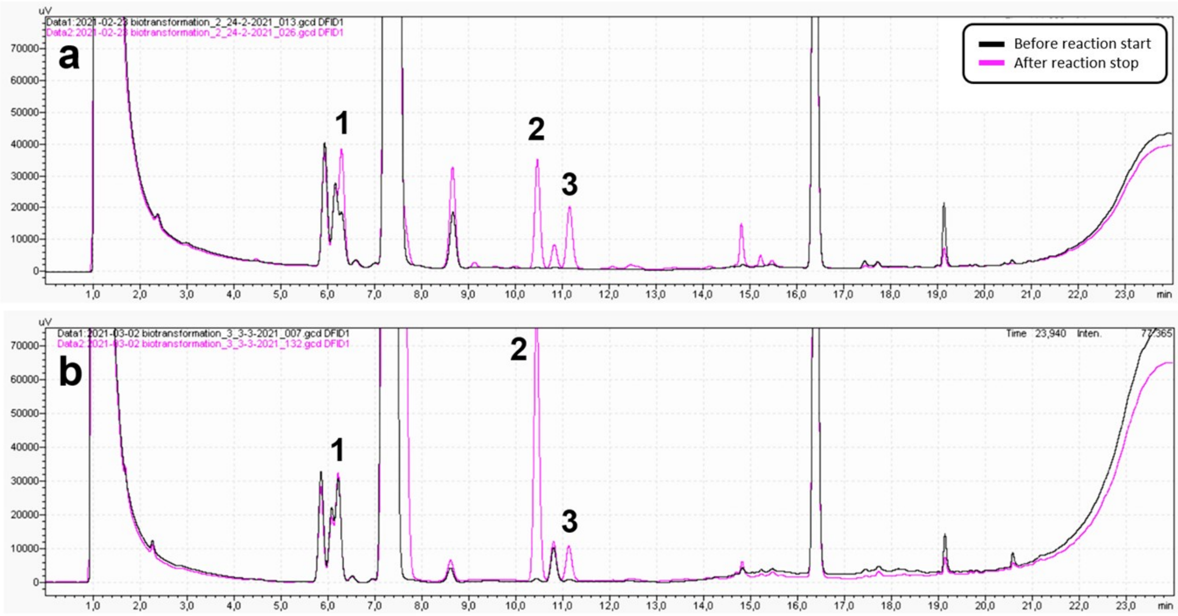

Figure S17. GC chromatograms of the epoxidation of *cis*-β-methylstyrene by imm-r*Aae*UPO. 1 = Benzaldehyde; 2 = (2*R*,3*S*)-2-methyl-3-phenyloxirane; 3 = Phenylacetone. (a) Using 20 mM h^{-1} ^{t}BuOOH as oxidant. The reaction mixture was analysed before start of the reaction (black) and after its stop at 4 h (pink). (b) Using 1 mM h^{-1} ^{t}BuOOH as oxidant. The reaction mixture was analysed before start of the reaction (black) and after its stop at 72 h (pink).

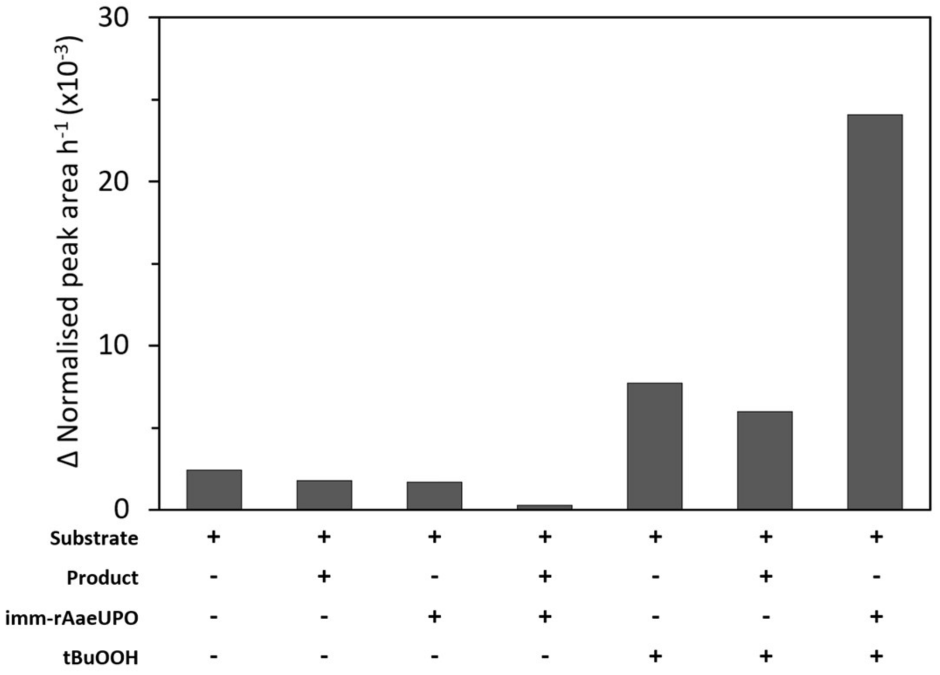

Figure S18. Control experiments to determine influencing factors of side product formation. Different components of the reaction mixture were incubated for 24h and the change of normalised peak area h^{-1} of all observed side products was determined. Substrate = *cis*-β-methylstyrene; Product = (2*R*,3*S*)-2-methyl-3-phenyloxirane (10 mM); [imm-r*Aae*UPO] = 0.5 µM; [^{t}BuOOH] = 10 mM h^{-1}.

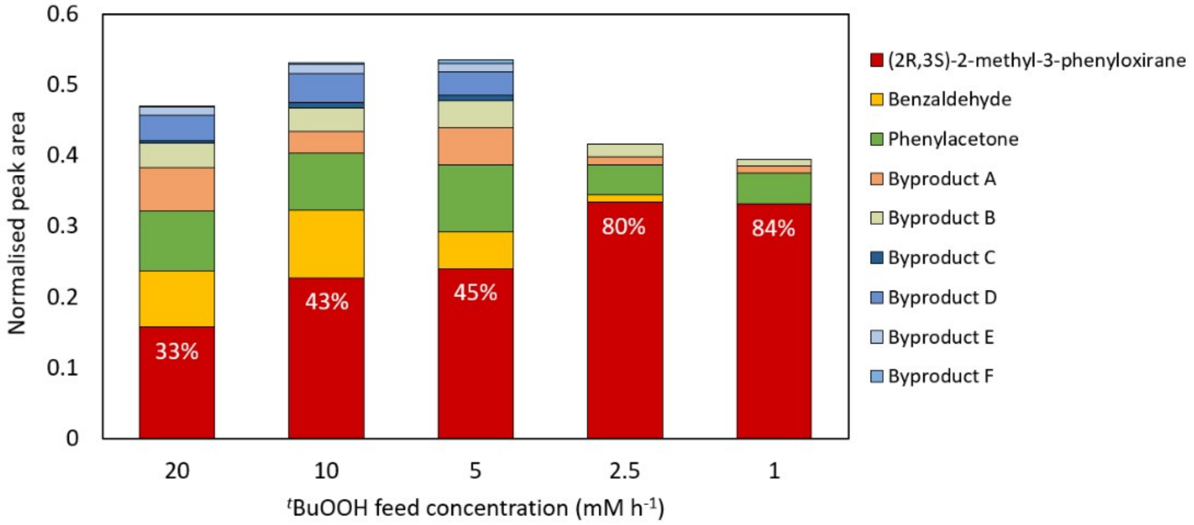

Figure S19. Product spectrum based on normalised peak area after reaction stop of the epoxidation of *cis*-β-methylstyrene by imm-r*Aae*UPO at different ^{t}BuOOH feed concentrations. The share of epoxide product is indicated in white letters. Reactions were analysed at the following time points: after 4h (20 mM h^{-1}), after 5h (10 mM h^{-1}), after 22h (5 mM h^{-1}) after 26h (2.5 mM h^{-1}), after 72h (1 mM h^{-1}).

S2.4 Epoxidation of styrene

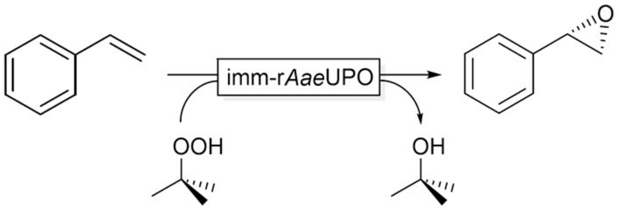

Scheme S1. Reaction equation for the epoxidation of styrene by imm-r*Aae*UPO with *tert*-butyl hydroperoxide as oxidant.

S2.4.1 Overview of the biotransformation results

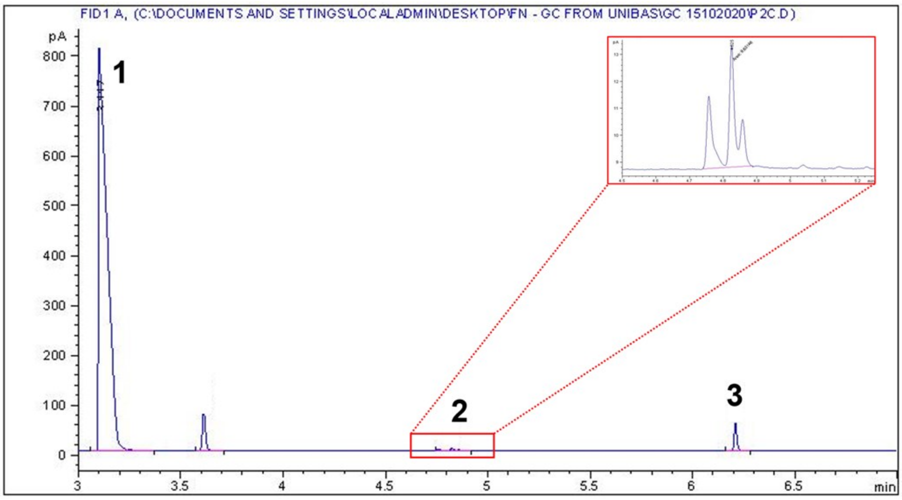

Figure S20. Example GC chromatogram for the epoxidation of styrene. Here, the reaction catalysed by imm-r*Aae*UPO is shown. 1 = Styrene; 2 = Product mix; 3 = Tetradecane (internal standard).

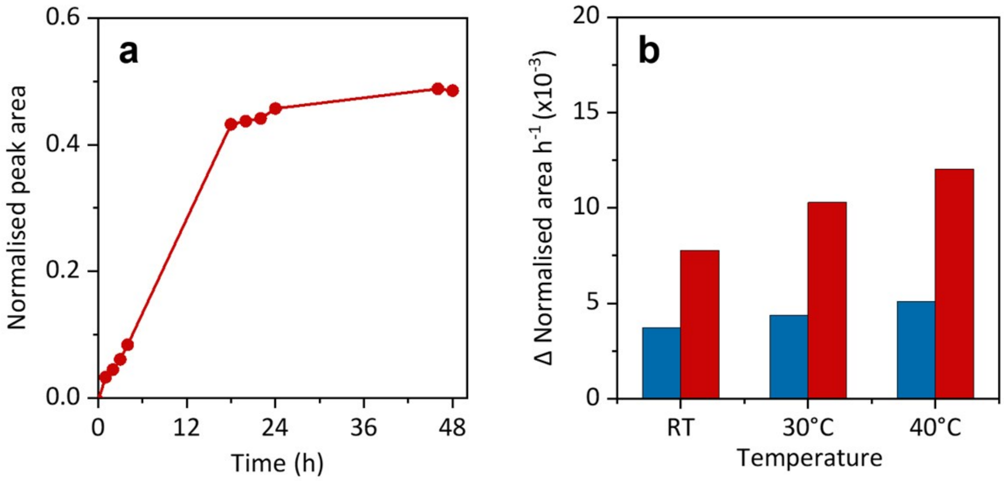

Figure S21. (a) Product formation over 48 h during epoxidation of styrene by imm-r*Aae*UPO with continuous ^{t}BuOOH feed. General reaction conditions: [r*Aae*UPO] = 0.8 μM, ^{t}BuOOH feeding rate = 8.5 mM h^{-1}, room temperature, shaking at 99 rpm with 60° angle in an overhead rotator. Data represented an average of duplicates. (b) Product formation rates over 1.5 h of free r*Aae*UPO (blue) and imm-r*Aae*UPO (red) for the epoxidation of styrene. General reaction conditions: [r*Aae*UPO] = 0.2 μM; ^{t}BuOOH pulse feeding of 1.25 mM every 15 min; temperatures were room temperature (RT), 30 °C, and 40 °C; shaking at 99 rpm with 60° angle in an overhead rotator.

S2.4.2 Influencing factors for the catalytic performance of imm-r*Aae*UPO

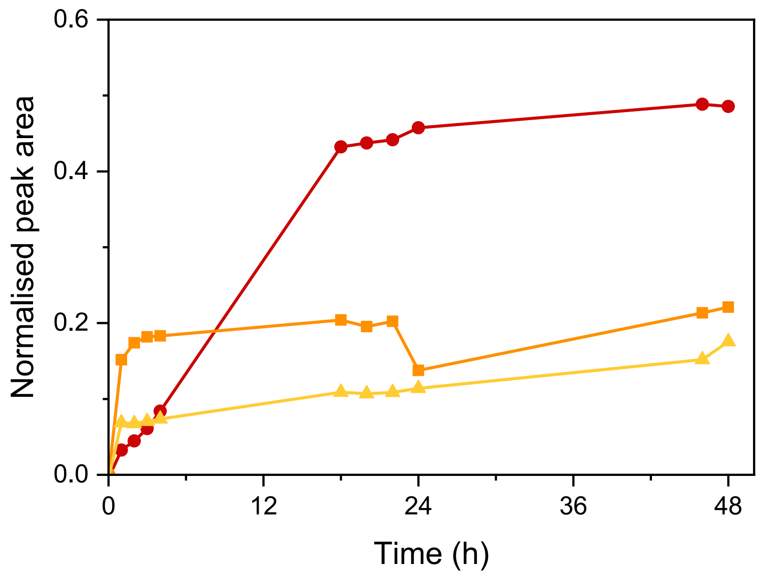

Figure S22. **Impact of ^{t}BuOOH feed rate.** Product formation over time for the epoxidation of styrene with different ^{t}BuOOH feed rates. Red circles = continuous ^{t}BuOOH feed at 8.5 mM h^{-1}, orange squares = 205 mM ^{t}BuOOH supply every 24 h, yellow triangles = 2.2 M ^{t}BuOOH (the equimolar amount of used styrene) at the beginning of the reaction. General reaction conditions: [r*Aae*UPO] = 0.8 µM, room temperature, shaking at 99 rpm with 60° angle in an overhead rotator.

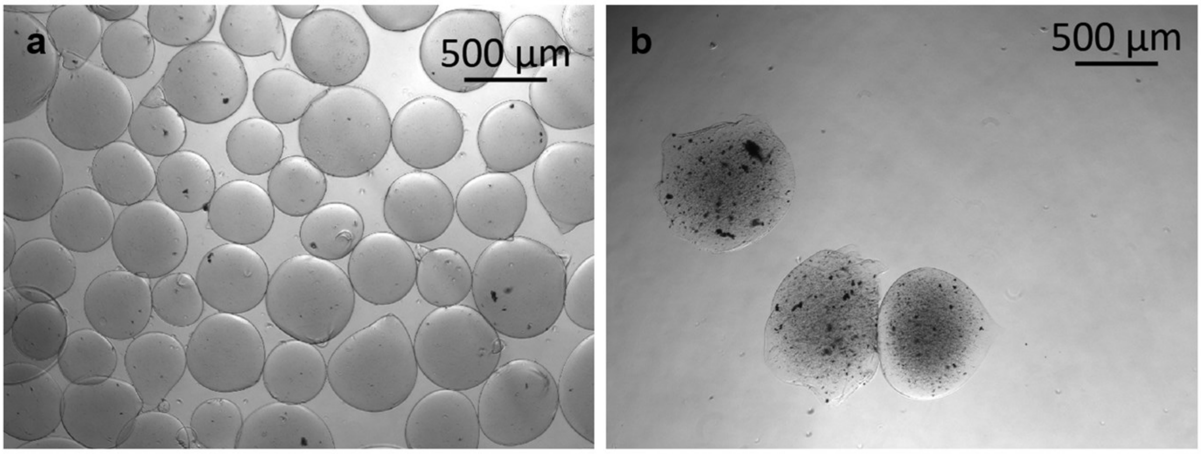

Figure S23. Appearance of imm-r*Aae*UPO before and after the epoxidation reaction. (a) Freshly prepared imm-r*Aae*UPO alginate beads. (b) imm-r*Aae*UPO after 72 h incubation in the reaction mixture containing neat styrene, ^{t}BuOOH and epoxidation products.

S3. Supporting comparative table

Table S6. Comparison of the catalytic performance of the proposed system with some methods reported.

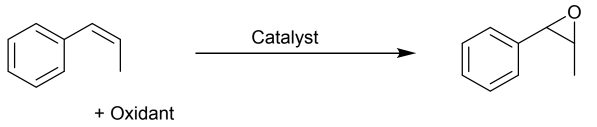

Catalyst	Oxidant	TN (Cat)[a]	Ref
$Co_{1,3}O_{1,4}$-N/C	tBuOOH	25<br>(150 after several recycles)	[12]
CYP101B1	$NADPH/O_2$ [b]	~3080[b]	[13]
StyAB[b]	$NADPH/O_2$[c]	2867	[14]
SMO	$NADPH/O_2$[d]	72 g g^{-1}[d]	[15]

[a] TN = $moles_{Product} \times moles_{Catalyst}^{-1}$;
[b] recombinant cells (*E. coli* overexpressing the P450 monooxygenase CYP101B1, from a *Novosphingobium* strain) were used as catalyst; the enzyme concentration was estimated to be 650 nM; O_2 was reductively activate by the P450 monooxygenase;
[c] StyAB: Styrene monooxygenase from *Pseudomonas* sp. VLB120 (recombinantly expressed in *E. coli*);
[d] SMO: Styrene monooxygenase from *Rhodococcus* sp. ST-10 styrene monooxygenase, recombinant *E. coli* cells were used as biocatalyst, the SMO concentration was not determined, therefore, the amount of mass of product per mass of whole cell biocatalyst is shown.

S4. References

[1] P. Molina-Espeja, S. Ma, D. M. Mate, R. Ludwig, and M. Alcalde, "Tandem-yeast expression system for engineering and producing unspecific peroxygenase," *Enzyme Microb. Technol.*, vol. 73–74, pp. 29–33, 2015.

[2] E. Fernández-Fueyo, Y. Ni, A. Gomez Baraibar, M. Alcalde, L. M. van Langen, and F. Hollmann, "Towards preparative peroxygenase-catalyzed oxyfunctionalization reactions in organic media," *J. Mol. Catal. B Enzym.*, vol. 134, pp. 347–352, 2016.

[3] J. B. Schenkman and I. Jansson, "Spectral analyses of cytochromes P450.," *Methods Mol. Biol.*, vol. 320, pp. 11–18, 2006.

[4] R. Y. O. Sato, "The Carbon of Liver," *J. Biol. Chem.*, vol. 239, no. 7, pp. 2379–2385, 1964.

[5] M. Hobisch *et al.*, "Solvent-Free Photobiocatalytic Hydroxylation of Cyclohexane," *ChemCatChem*, vol. 12, no. 16, pp. 4009–4013, 2020.

[6] W. Lu, Y. Shen, A. Xie, and W. Zhang, "Preparation and protein immobilization of magnetic dialdehyde starch nanoparticles," *J. Phys. Chem. B*, vol. 117, no. 14, pp. 3720–3725, 2013.

[7] J. Yu, P. R. Chang, and X. Ma, "The preparation and properties of dialdehyde starch and thermoplastic dialdehyde starch," *Carbohydr. Polym.*, vol. 79, no. 2, pp. 296–300, 2010.

[8] J. D. Pédelacq, S. Cabantous, T. Tran, T. C. Terwilliger, and G. S. Waldo, "Engineering and characterization of a superfolder green fluorescent protein," *Nat. Biotechnol.*, vol. 24, no. 1, pp. 79–88, 2006.

[9] M. C. R. Rauch, F. Tieves, C. E. Paul, I. W. C. E. Arends, M. Alcalde, and F. Hollmann, “Peroxygenase-Catalysed Epoxidation of Styrene Derivatives in Neat Reaction Media,” *ChemCatChem*, vol. 11, no. 18, pp. 4519–4523, 2019.

[10] R. A. Sheldon, “Metrics of Green Chemistry and Sustainability: Past, Present, and Future,” *ACS Sustain. Chem. Eng.*, vol. 6, no. 1, pp. 32–48, 2018.

[11] M. Hobisch, D. Holtmann, P. Gomez de Santos, M. Alcalde, F. Hollmann, and S. Kara, “Recent developments in the use of peroxygenases – Exploring their high potential in selective oxyfunctionalisations,” *Biotechnol. Adv.*, no. August, p. 107615, 2020.

[12] D. Banerjee, R. V. Jagadeesh, K. Junge, M.-M. Pohl, J. Radnik, A. Brückner and M. Beller, *Angew. Chem. Int. Ed.*, 2014, **53**, 4359-4363.

[13] M. R. Sarkar, J. H. Z. Lee and S. G. Bell, *ChemBioChem*, 2017, **18**, 2119-2128.

[14] K. Hofstetter, J. Lutz, I. Lang, B. Witholt and A. Schmid, *Angew. Chem. Int. Ed.*, 2004, **43**, 2163-2166.

[15] H. Toda, R. Imae and N. Itoh, *Tetrahedron Asymm.*, 2012, **22-23**, 1542-1549.
